# Identification of Heme Oxygenase-1 as a Putative DNA-Binding Protein

**DOI:** 10.3390/antiox11112135

**Published:** 2022-10-28

**Authors:** Alejandro Scaffa, George A. Tollefson, Hongwei Yao, Salu Rizal, Joselynn Wallace, Nathalie Oulhen, Jennifer F. Carr, Katy Hegarty, Alper Uzun, Phyllis A. Dennery

**Affiliations:** 1 Department of Molecular Biology, Cell Biology & Biochemistry, Division of Biology and Medicine, Brown University, Providence, RI 02912, USA; 2 Department of Pathology and Laboratory Medicine, Rhode Island Hospital, Providence, RI 02903, USA; 3 Center for Computational Biology of Human Disease, and Center for Computation and Visualization, Brown University, Providence, RI 02906, USA; 4 Department of Pediatrics, Warren Alpert Medical School of Brown University, Providence, RI 02903, USA; 5 Department of Pediatrics, Women and Infants Hospital, Providence, RI 02905, USA; 6 Center for Computational Molecular Biology, Brown University, Providence, RI 02906, USA

**Keywords:** heme oxygenase-1, DNA binding, proteinarium, 3D structural modeling

## Abstract

Heme oxygenase-1 (HO-1) is a rate-limiting enzyme in degrading heme into biliverdin and iron. HO-1 can also enter the nucleus and regulate gene transcription independent of its enzymatic activity. Whether HO-1 can alter gene expression through direct binding to target DNA remains unclear. Here, we performed HO-1 CHIP-seq and then employed 3D structural modeling to reveal putative HO-1 DNA binding domains. We identified three probable DNA binding domains on HO-1. Using the Proteinarium, we identified several genes as the most highly connected nodes in the interactome among the HO-1 gene binding targets. We further demonstrated that HO-1 modulates the expression of these key genes using *Hmox1* deficient cells. Finally, mutation of four conserved amino acids (E215, I211, E201, and Q27) within HO-1 DNA binding domain 1 significantly increased expression of Gtpbp3 and Eif1 genes that were identified within the top 10 binding hits normalized by gene length predicted to bind this domain. Based on these data, we conclude that HO-1 protein is a putative DNA binding protein, and regulates targeted gene expression. This provides the foundation for developing specific inhibitors or activators targeting HO-1 DNA binding domains to modulate targeted gene expression and corresponding cellular function.

## 1. Introduction

Heme oxygenase-1 (HO-1) is the rate-limiting enzyme of heme metabolism, which catalyzes the degradation of the heme into biliverdin, iron and carbon monoxide. It is a stress-inducible protein and one of the most ubiquitous and highly conserved antioxidant enzymes. A myriad of functions have been attributed to HO-1 as it plays important roles in maintaining cell homeostasis, tumorigenesis, immune function, reducing oxidative damage, and modulating both cell proliferation and apoptosis via regulation of intracellular levels of heme and heme metabolites [1,2,3,4]. Interestingly, HO-1 is also able to modulate cell function independently of its enzymatic activity by binding with itself, its isoenzyme HO-2, and caveolin-1 through protein–protein interactions [5]. As an example, HO-1 activity is negatively modulated by caveolin-1, by binding to a specific five-amino-acid long sequence in caveolin-1. Hyperoxia was shown to induce the physical interaction between caveolin-1 and HO-1 in fibroblasts, which reduced HO enzymatic activity [6]. As a compensatory response, oxidative stress led to activation of the antioxidant response element nuclear factor erythroid 2-related factor 2 (Nrf2), which induces HO-1, resulting in protection against reactive oxygen species. HO-1 upregulation in hyperoxia protected against lung injury in ventilated patients [7]. Using an integrated analysis of transcriptomic and proteomics, HO-1 was demonstrated to bind to key proteins, including gelsolin, Stat3 and Hspb1, which are associated with DNA and chromatin, RNA metabolism (post-transcription, including splicing), actin and cytoskeleton proteins in prostate cancer cells [8]. Therefore, HO-1 plays an important role in regulating its own enzymatic activity, other signaling pathways and cellular functions, through protein–protein interactions.

HO-1 can migrate to various cellular compartments, including the endoplasmic reticulum, mitochondria, and the nucleus [9,10]. Interestingly, when HO-1 migrates to the nucleus, it loses its enzymatic function [9,11]. We have previously demonstrated that HO-1 can be truncated at its c-terminal end to untether itself from the smooth endoplasmic reticulum and enter the nucleus [12], while others have shown that the enzyme signal peptide peptidase cleaves HO-1 at its transmembrane segment. This enzymatic cleavage releases HO-1 from the smooth endoplasmic reticulum, allowing its nuclear entry [13]. We have also reported that cleaved, nuclear HO-1 has limited catalytic activity but significant transcriptional regulatory activity by binding to transcription factors [12]. Within the nucleus, HO-1 is known to bind to Nrf2, Stat3, and other DNA repair proteins and to regulate transcriptional activity [12]. Furthermore, lysine residues of truncated HO-1 can be acetylated, allowing its binding to the Jun D subunit of the transcription factor AP-1. This post-translational modification is required to induce cell proliferation, migration, invasion, and metastasis [14]. The mechanisms by which HO-1 modulates transcriptional activity and gene expression are unclear. To our knowledge, no one has shown that HO-1 can directly bind to DNA.

The interaction of DNA-binding proteins to DNA can be non-specific, or can occur through a designated DNA binding domain that aligns to a highly specific and conserved DNA sequence called a DNA motif. Transcription factors are regulatory proteins whose function is to activate or inhibit transcription of DNA by binding to specific DNA sequences. Transcription factors have defined DNA-binding domains with up to 106-fold higher affinity for their target sequences than for the remainder of the DNA strand. These highly conserved sequences have been used to categorize the known transcription factors into various “families,” such as the MADS box-containing proteins, SOX proteins, and POU factors [15]. Transcription factors can also be classified by their three-dimensional protein structure, including basic helix-turn-helix, helix-loop-helix, and zinc finger proteins. These various structural motifs result in transcription factor specificity for the consensus sequences to which they bind. Although the protein structure of HO-1 does not contain a zinc finger, it consists of seven α-helices that may provide a pocket for DNA-binding [16,17]. To analyze whether this is in fact the case, we performed HO-1 chromatin immunoprecipitation sequencing (ChIP-Seq) using a mouse lung epithelial cell line. We further studied the 3D structure of HO-1 protein to better analyze how it binds to DNA. We then conducted a network visualization using a novel multi-sample, protein–protein interaction (PPI) tool, Proteinarium, to obtain information about the PPI network. We also used state-of-the-art 3D modeling tools including the ModelX and FoldX Suite for additional confirmation of binding interactions and binding sites on the HO-1 protein [18,19,20]. We then investigated the effect of HO-1 on the target genes in *Hmox1* deficient cells. Finally, we mutated conserved amino acids that were identified in HO-1 DNA-binding domain 1 (DBD-1), and measured target gene expression.

## 2. Material and Methods

### 2.1. MLE-12 Cell Culture

MLE-12 mouse lung epithelial cells (ATCC, Manassas, USA) were grown in Dulbecco’s medium:Ham’s F12 (50:50 mix) with 2% fetal bovine serum, 1% penicillin and streptomycin, 10 nM hydrocortisone, 0.005 mg/mL insulin, 0.01 mg/mL transferrin, 30 nM sodium selenite, and 10 nM β-estradiol. Cells were kept at 37 °C under 21% O_2_ and 5% CO_2_ in a humidified chamber. The cells for ChIP assay were grown in 15 cm^2^ culture plates and collected at 75% confluency.

### 2.2. CRISPR/Cas9 Deficient of HO-1 in MLE-12 Cells

A two-step CRISPR system (Sigma-Aldrich, St. Louis, MO, USA) was used as per the manufacturer’s instructions. The Cas9-blasticidin (LVCAS9BST) lentivirus was transduced into MLE-12 cells followed by blasticidin selection. Cells were then transduced with a non-coding-gRNA lentivirus and a HO-1-gRNA (TATGTAAAGCGTCTCCACG) puromycin^+^ lentivirus. Cells were then selected with puromycin and single-sorted into 96-well plates using flow cytometry. Deletion efficiency was determined by measuring HO-1 protein levels using Western blot.

### 2.3. Transcriptional Analysis

qRT-PCR assays were performed using the ThermoFisher Taqman qPCR master mix and Taqman fluorescent probes. The qPCR assays were done with 18s (Taqman Hs99999) as a housekeeping control in an ABI5000 instrument. Probes used were Ubc (Mm01198158_m1), Dnajb1 (Mm00444519_m1), Eef1a1 (Mm01973893_g1), Rpl27 (Mm00821100_g1), Uba52 (Mm02601856_g1), Hmgb2 (Mm07307431_s1), Hnrnpa2b1 (Mm01332941_m1), Eif1 (Mm00783932_s1), E2f1 (Mm00432939), E2f4 (Mm00514160_m1), Nr1d1 (Mm00520711), Malat1 (Mm01227912_s1), Nr1d1 (Mm00520711), Foxa1 (Mm00484713_m1), Bola1 (Mm01255885_g1), Coq9 (Mm00804236_m1), Lias (Mm00522477_m1), Fzd2 (Mm02524776_s1), Gtpbp3 (Mm00466666_m1), Rpl36 (Mm03030414_g1), and Eif1 (Mm00783932_s1).

### 2.4. ChIP-Seq Assay

**Figure 1:**
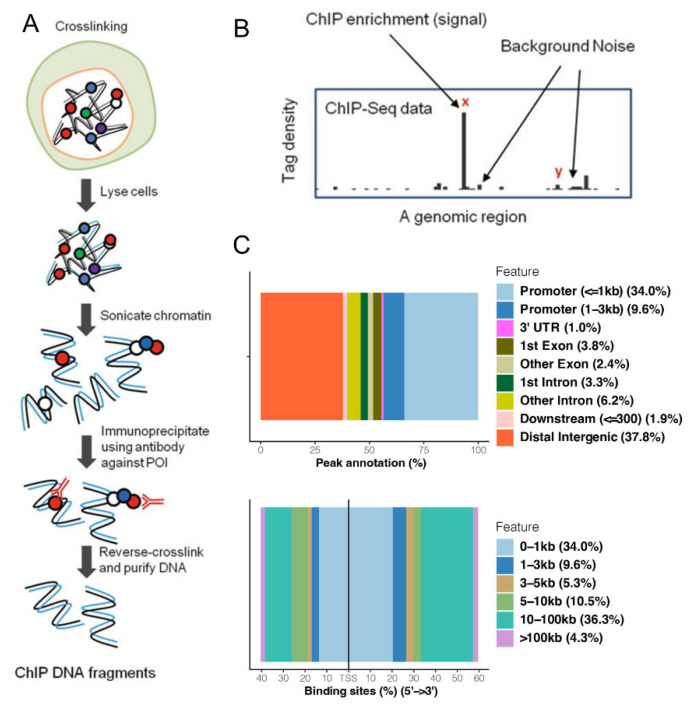
HO-1 ChIP-seq flowchart and binding to DNA. (**A**) Protocol for ChIP-seq. (**B**) Meaning of enriched peaks, or hits, in ChIP-seq data. (**C**) HO-1 DNA peak locations (distribution of peaks inside genomic features (top) or peak distance from transcription start site (bottom)) showing that majority of HO-1-specific peaks are either less than 1 KB from a promoter/Transcriptional Start Sites (TSS) or in distal intergenic regions (10–100 kb from promoter/TSS).

The ChIP-Seq experiment was adapted from previously described [21]. MLE-12 cells were incubated with 1% formaldehyde for 10 min for crosslinking. After washing with wash buffer (10 mM HEPES, 10 mM EDTA, 0.5 mM EGTA, 0.25% Triton X-100, 0.2 mM PMSF, and 1 × protease inhibitors), the cells were counted and aliquoted into 100 × 10^6^ cells per sample. Cells were then sonicated to shear chromatin using lysis buffer (1% SDS, 20 mM HEPES, 10 mM EDTA, and 50 mM NaCl) in a Covaris S220 High Performance Ultrasonicator. To confirm that the DNA shearing process was successful, the DNA was incubated with RNAseA (Thermo Scientific, EN0531, Waltham, MA, USA) and reverse-crosslinked overnight at 65 °C followed by proteinase K incubation (Thermo Scientific, EO0491), phenol chloroform extraction, and ethanol precipitation. The clean DNA was run on a fragment analyzer to confirm desired shearing around 200 bp in length. The sheared chromatin was then incubated with HO-1 antibody (Abcam, ab13243, Waltham, MA, USA) or IgG (Abcam, ab171870) overnight with dynabeads A/G beads (Thermo Scientific, 78609). The beads were magnetically isolated and washed with RIPA buffer (50 mM TRIS, 1% NP-40, 2 mM EDTA, 0.1% sodium deoxycholate, 0.1% SDS, 300 mM NaCl) and DNA was isolated as per manufacturer’s instructions. The ChIP-seq library was generated by Genewiz (Genewiz, South Plainsfield, NJ, USA) using Truseq ChIP library generation kit and sequenced with Illumina Hi-Seq 2 × 150 bp single index per lane with an average of 58 million reads per sample (Figure 1A,B).

### 2.5. Computational Analysis of ChIP-Seq and Motif Binding

Analysis of ChIP-seq data was adapted from Zhang, 2008 [22]. After acquiring the raw data and uploading to the server, quality control was performed as follows. In short, raw and trimmed reads were loaded and analyzed for quality control using FastQC (V0.11.5) (Supplementary Tables S1–S3, Supplementary Figures S1–S3). Reads were trimmed to remove adapters, and low-quality or short sequencing with Trimmomatic (V0.36) [23] ran in PE mode (“ILLUMINACLIP:~/trimmomatic-0.36/adapters/TruSeq3-PE-2.fa:2:30:5:6:true”:“SLIDINGWINDOW:10:25 MINLEN:75”). Reads were aligned to Ensembl (GRCm38.p6) with bwa (V0.7.15) and further downstream data manipulation was performed using Samtools (V1.9) [24]. Duplicate reads were marked (MarkDuplicates) and alignment summary statistics (CollectAlignmentSummaryMetrics, CollectWGSMetrics) were generated using Picard tools (V2.9.2) (Supplementary Tables S4–S6). Peaks were called using MACS2 callpeak (V 2.1.2) with the input samples as control for IgG and HO-1 samples, and a nominal *p*-value cutoff of 1 × 10^−3^.

Further downstream analysis was performed in R 3.6.3 (R Core Team (2020). R: A language and environment for statistical computing. R Foundation for Statistical Computing, Vienna, Austria). Non-specific peaks from the IgG libraries were filtered out using findOverlapsOfPeaks from the ChIPPeakAnno (V 3.20.1) package [25,26]. The resulting peaks were annotated using annotatePeak from the ChIPseeker (V 1.22.1) package [27]. The distribution of peaks across promoters, exons, introns, UTRs, and distal or intergenic regions were visualized using ggplot2 (V 3.3.0 H. Wickham. ggplot2: Elegant Graphics for Data Analysis. Springer-Verlag New York, 2016.) and the annoStat slot from the annotatePeak results. The distribution of peaks relative to transcription start sites were visualized using ChIPseeker plotDistToTSS function [27].

All of the code for computational analysis of ChIP data (alignment, read and alignment QC, read trimming, and downstream analyses in R) is available on GitHub (https://github.com/compbiocore/Scaffa, accessed on 1 September 2022).

Hit mapped reads were further analyzed for their location in the genome using in-house scripts and using data from the UCSC human genome browser [28], gene ontology was observed using gclusterProfiler to run enrichment analysis (enrichKEGG function), and common motifs of the reads were found de novo using the MEME suite (MEME Suite Software) [29]. Statistical analyses such as *t*-tests and one-way ANOVA were ran using Prism 9.4.0 (GraphPad Software, San Diego, CA, USA).

### 2.6. Performing Protein–Protein Interaction (PPI) Networks (Proteinarium)

We ran Proteinarium [18] on the genes shown to bind HO-1 by ChIP-Seq and on the genes identified through our BLAST search [30] using the results of the MEME motif search [31]. Proteinarium is a PPI network visualization and analysis tool. Proteinarium exclusively accepts human gene identifiers so we converted mouse gene names to HGNC (HUGO Gene nomenclature committee) symbols prior to analysis [32]. Out of 118 genes identified by ChIP-Seq, we excluded 48 genes which did not carry either official names or interaction information. Proteinarium was run using default parameters and by setting the maximum path length to two nodes of separation. Here, the maximum path length parameter defines the number of imputed proteins which may link seed genes using experimentally validated protein–protein interaction data.

### 2.7. Functional Enrichment

We performed Gene Ontology (GO) Term [33] and Kyoto Encyclopedia of Genes and Genomes (KEGG) [34] pathway enrichments using the Functional Annotation Tool provided by the Database for Annotation, Visualization and Integrated Discovery (DAVID) v6.8 [35,36]. Bubble charts of significantly enriched Biological Process GO Terms and their associated False Discovery Rate corrected *p* values were plotted using the ggplot2 v3.3.2 R package.

### 2.8. Predictive Modeling of Apo HO-1 dsDNA Binding Complex Structures

We developed a novel workflow for predictive structural modeling of the dsDNA binding complex of human apo HO-1 and characterization of genomic binding targets of human apo HO-1 using ModelX [19], FoldX [37], UCSF Chimera [38], Linux, and R scripting. The complete workflow consists of three main steps: (1) all possible dsDNA binding sites and dsDNA backbone conformations on HO-1 were identified using ModelX software; (2) the predicted set of all possible dsDNA backbone binding fragments to a set of continuous and highly energetically favorable dsDNA-peptide complexes in three distinct locations on the 3D structure of HO-1 were refined using FoldX software; and (3) the specific dsDNA nucleotide sequences within each dsDNA backbones which were most energetically favorable to bind the peptide residues in each putative DNA binding domain were identified using FoldX software. Finally, we showed the molecular binding interactions between each of the three DNA binding domains and their compatible double stranded DNA molecules using the visualization program Chimera. We have described our workflow in detail below.

### 2.9. ModelX- dsDNA-Protein Docking

We used the ModelX tool suite for biomolecular modeling to identify short dsDNA backbone structures which are compatible binding partners with residues in a query Protein Data Bank (PDB) protein file. We selected the human heme-free apo HO-1 protein PDB structure which we extracted from the PBD file (PDB ID = 1N16) as a query protein structure since it has been previously shown that the heme binding C-terminus domain is cleaved from HO-1 before it’s transportation to the nucleus [12]. We used selective filters to find many short dsDNA helix backbone fragments which are predicted to bind HO-1 with high confidence and assembled a cloud of all predicted fragments positioned around the HO-1 structure in PDB format. We then searched for continuous stretches of short dsDNA backbones in the model to identify extended fragments in order to further refine our set of predicted dsDNA binding backbones. By identifying backbone sequences with greater lengths, we were able to identify binding sequences contained within the modeled dsDNA helix backbones with higher specificity since they provide more points of comparison.

### 2.10. Identification of Compatible dsDNA Binding Targets of Apo HO-1

We performed a series of energy calculations using the DNAScan tool from the FoldX Suite [21] to predict the most probable nucleotide sequence contained within each extended dsDNA backbone fragment. Using the energy file output of DNAScan, we assembled energy matrices containing the change in Gibbs Free Energy (ΔΔG) of the HO-1 structure incurred by mutating each base along each of the predicted binding fragments to each of the four nucleotides. We used these energy matrices to identify the most probable dsDNA sequences contained in each of the dsDNA backbone fragments by selecting the nucleotides in each fragment position which produced the most stable binding complex structure with the lowest free energy.

We used the DNAContact FoldX tool [21] to identify the positions along each dsDNA fragment which make direct contact with one or more residues on the HO-1 structure. We only considered fragments which had at least three DNA contact positions for further analysis.

To identify compatible dsDNA genomic binding targets of HO-1, we used these newly identified energetically favorable binding sequences to thread the genomic sequence of our genes of interest. We only considered positions within the dsDNA fragments which were shown to directly bind HO-1 by the results from FoldX DNAContact. Since our calculated energetically favorable binding sequences are double stranded, we simultaneously threaded both the genomic sequence and its complementary sequence to identify compatible binding sequences.

We identified all compatible genomic binding positions in our target genes and recorded the coordinates as well as total number of binding sites per gene. We identified genes which are predicted to have the highest binding affinity to HO-1 by normalizing the total number of compatible HO-1 binding sites on each gene by the gene length. We characterized the functions of the genes with the greatest number of compatible binding sites using DAVID functional enrichment tools. We performed sequence threading using the sequences of the genes in the gene lists produced by CHIP-seq and by our subsequent motif search and have summarized this data in several tables (Supplementary Tables S1 and S7).

### 2.11. Visualization of HO-1 Protein and dsDNA Binding Fragment PDB Data

We used UCSF Chimera program to perform all visualizations of dsDNA fragments and human apo HO-1 structural data in PDB format [21].

### 2.12. Transfection of Mutated HO-1 Plasmids into Hmox1 Deficient Cells

Four conserved amino acids, including E215, I211, E201, and Q27, in HO-1 DBD1 were mutated to alanine, which was validated by sequencing and cloned in the pcDNA3.1 vector. The wild-type or mutated HO-1 plasmids (2 μg) were transfected into *Hmox1* deficient MLE-12 cells for 24 h by using lipofectamine 3000 according to the manufacturer’s protocol. The cell pellets were collected for measuring expression of Fzd2, Gtpbp3, Rpl36, and Eif1 genes using the qRT-PCR.

### 2.13. Statistical Analysis

Experiments were repeated biologically three times, with three technical replicates for quantitative qRT-PCR assay. Data are expressed as mean ± standard error of the mean (SEM). The unpaired *t*-test was used for detecting statistical significance of the differences between means of two groups if the data are normally distributed using Graph Pad Prism 9 software. Welch-corrected t test was used if the data are not normally distributed. For multiple comparisons, the statistical significance of the differences was evaluated by using one-way ANOVA followed by Tukey’s post-test to specifically compare indicated groups. Statistical significance was considered when the *p* value was less than 0.05.

## 3. Results

### 3.1. CHIP-Seq Reveals That HO-1 Binds to DNA

ChIP-Seq is a robust method used to analyze protein interactions with DNA. Thus, we performed a ChIP-Seq in mouse lung epithelial MLE-12 cells to determine whether HO-1 could be a DNA-binding protein. The experiment yielded over 50 million 250 paired-end reads per sample. The data went through quality control, alignment, and further downstream analysis (Supplementary Tables S1 and S2, Supplementary Figures S1–S3). Compared to IgG control, hundreds of hits on 118 separate genes were observed in the HO-1 ChIP assay (Supplementary Table S7). These aligned to less than 1 kb from the promoter (33.97% of hits) and within the distal intergenic regions (37.8% of hits) (Figure 1A–C). The KEGG gene ontology analysis of the DNA hits revealed that the genes are part of pathways related to cellular responses to external stimuli, cellular responses to stress, cellular senescence, DNA replication pre-initiation, oxidative stress-induced senescence, programmed cell death, G1/S transition, assembly of the pre-replicative complex, metalloproteases DUBs, and apoptosis-induced DNA fragmentation (Supplementary Figure S4). Table 1 showed the top 10% of the 118 genes with highest statistical significance as well as a known relationship of the genes to HO-1 in the literature.

**Figure 2:**
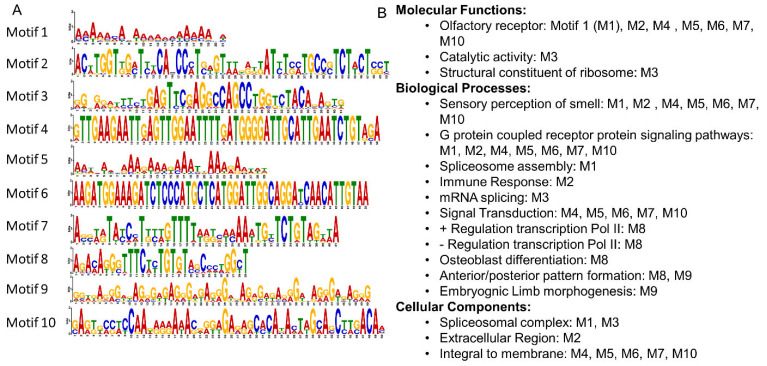
DNA sequence motifs shown as a sequence logo. (**A**) The 10 motifs were found de novo across the 118 hit genes found in HO-1 ChIP-seq. (**B**) Molecular functions, biological processes, and cellular components related to the motifs found in (**A**) when comparing those motifs to motifs found across the entire human genome.

The sequences from all 118 gene targets were submitted to motif analysis yielding 10 motifs that are common across subsets of our hits (Figure 2A). Although none of these motifs were a perfect match to known motif libraries, variations of these motifs were found to align to 130 genes including 45 genes that had multiple motifs within each gene. From these motifs we also ran function analysis leading to the molecular functions, biological processes, and cellular components in which the genes are known to play a role (Figure 2B). These results suggest that HO-1 protein putatively binds to DNA with a preference for specific motifs sequences.

### 3.2. PPI Network Analysis Reveals Multiple Interactions

**Figure 3:**
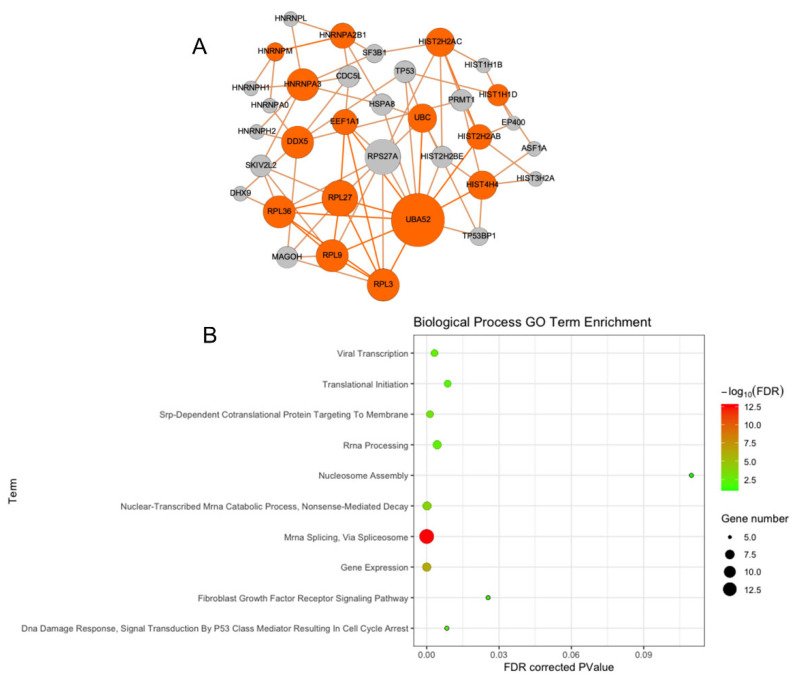
Protein–protein Interaction (PPI) network of the geneset. (**A**) PPI network of the gene set produced from the ChIP-Seq results were presented by using Multisample Network Analysis and Visualization Tool, Proteinarium. Orange color represents the proteins from the seed genes (input gene list). Gray color represents the imputed proteins from the interactome. (**B**) Top 10 biological process Gene Ontology terms enriched among the 34 genes found to exist in a PPI network provided by Proteinarium.

To get additional confirmation of binding interactions and binding sites, we analyzed the PPI network of the gene set produced from the ChIP-Seq results and produced a network visualization of the interactions by using the Proteinarium. We ran Proteinarium with the list of 70 human gene identifiers (ids) and generated a protein–protein interaction network that contains 34 genes. Of the 70 genes in the input list, 15 were found to interact in a network with 19 imputed genes based upon a maximum imputed path length of 2 nodes of separation. Using Proteinarium, we identified Uba52, Ubc, Hist2h2ab, Hist2h2ac, Ddx5, Eef1a1, Rpl3, Rpl9, Rpl27, Rpl36, and Hnrnpa3 as the most highly connected nodes in the interactome among the HO-1 gene binding targets identified by CHIP-seq (Figure 3A). These results suggest that there are multiple genes that interact with HO-1 protein.

### 3.3. Functional Enrichment of Protein–Protein Interaction Network Reveals Many Biological Processes Are Impacted by HO-1

Functional enrichment analysis of the 34 genes found to exist in the protein–protein interaction network yielded significantly enriched GO and KEGG pathway terms using a false discovery rate (FDR) corrected *p*-value threshold of 0.05 or lower. Top significantly enriched biological process GO terms among genes in the protein–protein interaction network included mRNA splicing via the spliceosome, gene expression, nuclear-transcribed mRNA catabolic processes, SRP-dependent cotranslational protein targeting, rRNA processing, DNA damage response, signal transduction by p53 class mediator resulting in cell cycle arrest, translational initiation, and fibroblast growth receptor signaling (Figure 3B). Top significantly enriched cellular component GO terms included the nucleoplasm, intracellular ribonucleoprotein complex, catalytic step 2 spliceosome, nucleus, nucleolus, nucleosome, cytosolic large ribosomal subunit, extracellular exosome, and nuclear chromatic. Two significantly enriched molecular function GO Terms were identified to be included poly(A) RNA binding and RNA binding. Additionally, the spliceosome KEGG pathway was identified to be significantly enriched. Altogether, these results indicate that HO-1 modulates gene expression involved in many biological processes.

### 3.4. Predictive Modeling of Apo HO-1 dsDNA Binding Complex Structures Reveals Several HO-1 DNA Binding Domains

**Figure 4:**
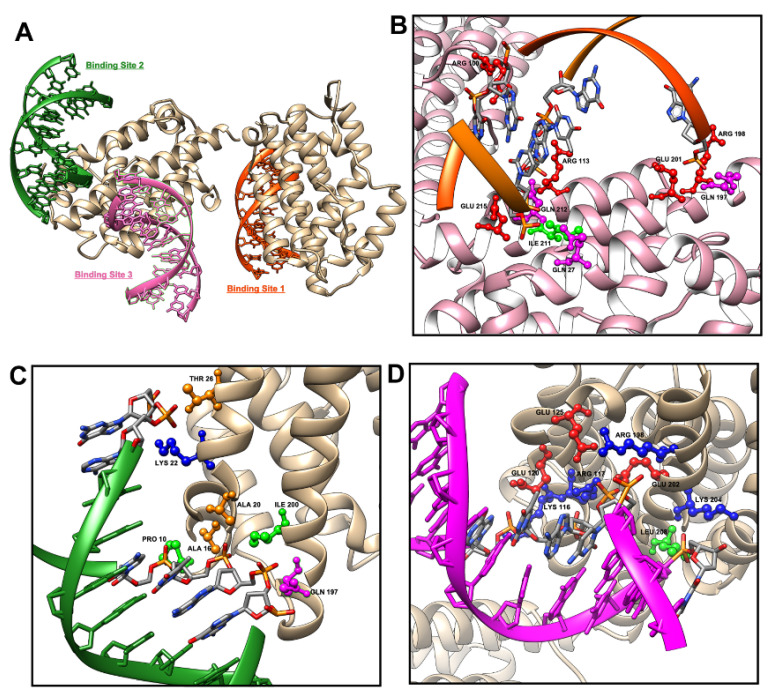
Predicted dsDNA binding sites on human apo HO-1 protein. (**A**) Three predicted dsDNA (10 bp binding fragments) binding sites on human apo HO-1 protein were presented. Binding site 1 was represented in orange color, binding site 2 was represented in green color and binding site 3 was represented in pink color. (**B**) Close view of binding site 1 contains 9 residues which contact with 7 DNA positions on a predicted dsDNA fragment. (**C**) Close view of binding site 2 contains seven residues which contact with 6 DNA positions on a predicted dsDNA fragment. (**D**) Close view of binding site 3 contains 8 residues which contact with 5 DNA positions on a predicted dsDNA fragment.

**Figure 5:**
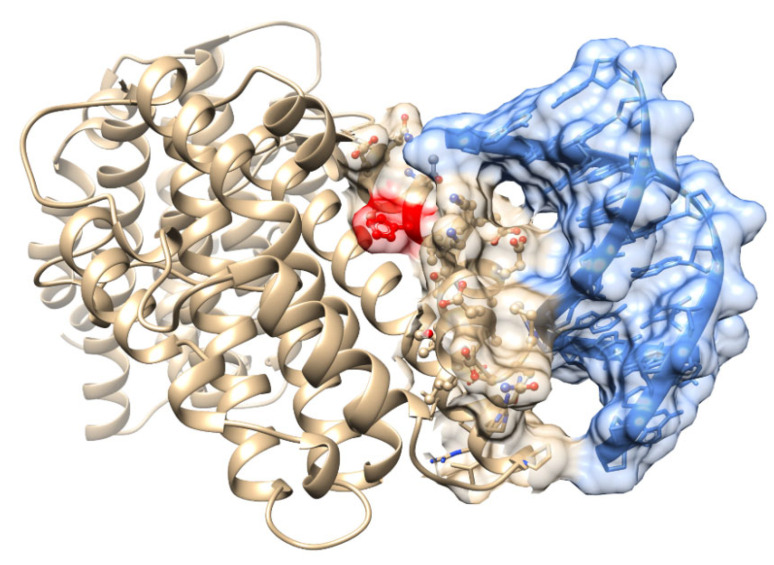
HO-1 DNA binding domain 2 is localized to the heme binding domain of HO-1. We presented that HO-1 DNA binding domain 2 (HO-1 DBD-2) contains peptide residues which surround the heme-binding functional ring of HO-1 on peptide residue His25 (red). The solvent-excluded molecular surfaces of the HO-1 peptide residue molecules within HO-1 DBD-2 were visualized as a semitransparent tan surface. The semitransparent transparent solvent-excluded molecular surfaces were used to show the 10 bp dsDNA molecule (blue) predicted to bind HO-1 DBD-2. dsDNA and HO-1 peptide residue contacts were illustrated where the molecular surface of the dsDNA (blue) merges with the molecular surface of the HO-1 DBD-2 peptide residues (tan).

To identify the predicted dsDNA binding domains on human apo heme-free HO-1, we used the workflow powered by ModelX and FoldX software. We identified four predicted dsDNA binding domains on human apo heme-free HO-1. We excluded one binding domain for which the predicted dsDNA fragment had only one DNA contact position. The other three binding sites were included for consideration as probable DNA binding domains on HO-1. Here, we have named these predicted binding domains HO-1 DBD-1, DBD-2, and DBD-3 (Figure 4). We presented our predictive characterization of these three human heme-free apo HO-1 DNA-binding domains (Figure 5). Summary and detailed information on the number of binding protein residues and nucleotides, specific compatible nucleotides and amino acids for each of the three DNA binding domains are described in detail in Table 2 and Table 3. Three probable DNA binding domains of HO-1 are identified.

### 3.5. Identification of Compatible dsDNA Binding Targets of Apo HO-1 among CHIP-Seq Genes

As shown in the link (https://docs.google.com/spreadsheets/d/1ewOX-wBOIg69V1DAs8Ua5HCDXALqsPFr/edit?usp=sharing&ouid=117066342160448117876&rtpof=true&sd=true, accessed on 1 September 2022), we identified targeted CHIP-seq genes that bind to HO-1 DNA-binding domains. The murine targets were converted to human targets since there is no published murine apo HO-1 protein structure. We describe the top 10 genes predicted to bind each HO-1 DNA-binding domain. Several of the genes with the highest HO-1 binding affinity, including UBA52, Rpl27, and Rpl36, are among the most connected nodes in the PPI network generated with Proteinarium.

#### 3.5.1. HO-1 DNA-Binding Domain 1 Targets among CHIP-Seq Genes

We found that 62 of 71 human genes which were found to bind HO-1 by ChIP-Seq and which are conserved between human and mice contain at least 1 dsDNA binding sequence which is compatible with the HO-1 DBD-1 binding domain. Of these 62 genes, Hmgb2, Fzd2, Gtpbp3, Rpl36, Ankle1, Mrpl12, Npm3, Particl, Eif1, and Ranbp1 were the top 10 genes with the greatest proportion of HO-1 DBD-1 compatible binding sites normalized by gene length.

#### 3.5.2. HO-1 DNA-Binding Domain 2 Targets among CHIP-Seq Genes

We found that 65 of 71 human genes which were found to bind HO-1 by ChIP-Seq and which are conserved between human and mice contain at least 1 dsDNA binding sequence which is compatible with the HO-1 DBD-2 binding domain. Of these 65 genes, Rpl27, Hmgb2, Ankle1, Mat2a, Dnajb1, Mrpl12, Npm3, Nfe2l3, Orc6, and Ranbp1 were the top 10 genes with the greatest proportion of HO-1 DBD-2 compatible binding sites normalized by gene length.

#### 3.5.3. HO-1 DNA-Binding Domain 3 Targets among CHIP-Seq gENE

We found that 68 of 71 human genes which were found to bind HO-1 by ChIP-Seq and which are conserved between human and mice contain at least 1 dsDNA binding sequence which is compatible with the HO-1DBD-3 binding domain. Of these 68 genes, Rn7sk, Npm3, Particl, Malat1, Etfrf1, Hmgb2, Sart3, Nkain2, Fut10, and Uba52 were the top 10 genes with the greatest proportion of HO-1 DBD-3 compatible binding sites normalized by gene length.

### 3.6. Identification of Compatible dsDNA Binding Targets of Apo HO-1 among Motif BLAST Hit Genes

As shown in the link (https://docs.google.com/spreadsheets/d/1ux5PoBpGpWJ5iaeuCpUf-PkFrrGGkE7x/edit?usp=sharing&ouid=117066342160448117876&rtpof=true&sd=true, accessed on 1 September 2022), we identified targeted Motif BLAST Hit genes that bind to HO-1 DNA-binding domains. The murine targets were also converted to human targets since there is no published murine apo HO-1 protein structure.

#### 3.6.1. HO-1 DNA-Binding Domain 1 Targets among Motif BLAST Hit Genes

We found that 99 of 100 human genes which were found to have close homology with DNA binding motifs identified from genes found to bind HO-1 by ChIP-Seq contain at least 1 dsDNA binding sequence which is compatible with the HO-1 DBD-1 binding domain. Of these 99 genes, Foxq1, Etv2, Ascl2, Foxc1, Foxc2, Sp5, Barx1, Sox4, and Maz were the top 10 genes with the greatest proportion of HO-1 DBD-1 compatible binding sites normalized by gene length.

#### 3.6.2. HO-1 DNA-Binding Domain 2 Targets among Motif BLAST Hit Genes

We found that 98 of 100 human genes which were found to have close homology with DNA binding motifs identified from genes found to bind HO-1 by ChIP-Seq contain at least 1 dsDNA binding sequence which is compatible with the HO-1 DBD-2 binding domain. Of these 99 genes, Sp5, Maz, Barx1, Foxa2, Foxa1, Ascl2, Lyl1, Rxra, Srf, and Evx1 were the top 10 genes with the greatest proportion of HO-1DBD-2 compatible binding sites normalized by gene length.

#### 3.6.3. HO-1 DNA-Binding Domain 3 Targets among Motif BLAST Hit Genes

We found that 99 of 100 human genes, which were found to have close homology with DNA binding motifs identified from genes found to bind HO-1 by ChIP-Seq, contain at least 1 dsDNA binding sequence compatible with the HO-1 DBD-3 binding domain. Of these 99 genes, Sry, Nr1d1, Tbx21, E2f4, Gli1, Sox2, Foxq1, Tbx3, Nanog, and Foxj2 were the top 10 genes with the greatest proportion of HO-1 DBD-3 compatible binding sites normalized by gene length.

### 3.7. Functional Enrichment of HO-1 Binding Gene Targets Reveals a Role in Regulation of Transcription Factor Regulation, Cell Proliferation, and Tissue Development

To understand the functional impact of predicted HO-1 binding on regulatory domains of the genes found to bind HO-1 by ChIP-Seq, we performed a functional enrichment analysis on the top 50% of genes which had a higher proportion of HO-1 binding sequences after normalization by gene length. We found that the top 50% of gene targets for all three DBDs had commonly shared significantly enriched biological functions. The top significantly enriched biological functions shared by the gene targets of all three HO-1 DBDs involved DNA transcription, regulation of transcription, and tissue development including hemopoiesis, regulation of cell proliferation and organogenesis (Supplementary Figure S4).

### 3.8. Hmox1 Deficient MLE-12 Cells Show Dysregulation of Multiple Target Genes

**Figure 6:**
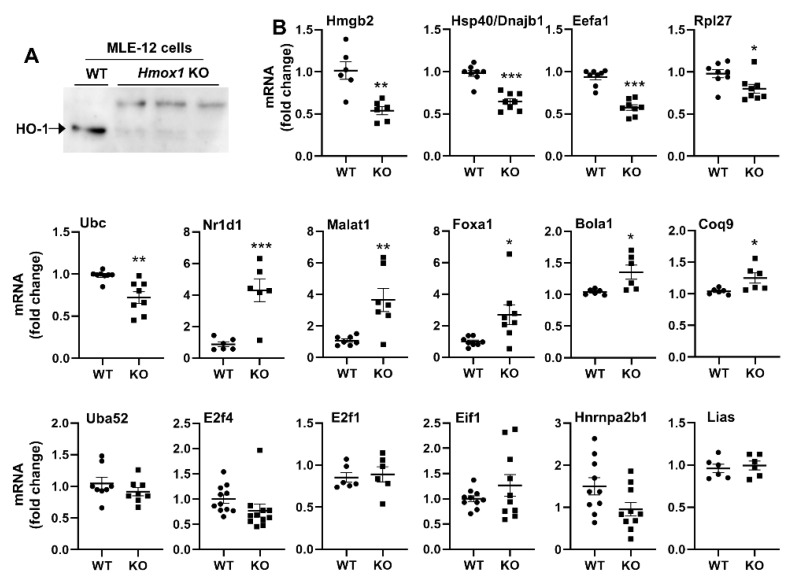
Gene expression in *Hmox1* deficient cells. (**A**) Western blot confirmed HO-1 deletion in *Hmox1* deficient MLE-12 cells. Each lane was loaded with 10 μg protein. (**B**) Transcriptional levels of targeted genes measured by qRT-PCR in *Hmox1* deficient (KO) and WT MLE-12 cells. Data are expressed as mean ± SEM. N = 6–8. * *p* < 0.05, ** *p* < 0.01, *** *p* < 0.001 vs. WT group.

In order to determine whether HO-1 had a direct effect on the transcription of identified genes, we generated a *Hmox1* deficient line using the CRISPR-Cas9 system. Using qRT-PCR, we showed that expression of several genes identified as binding to HO-1, namely DnaJ heat shock protein family Hsp40/Dnajb1 Member B1 (Hsp40), eukaryotic translation elongation factor 1 alpha 1 (Eefa1), ribosomal protein L27 (Rpl27), ubiquitin C (Ubc), and high-mobility group protein B2 (Hmgb2), was significantly downregulated in Hmox1 deficient MLE-12 cells (Figure 6, Supplementary Figure S5). In contrast, the expression of nuclear receptor subfamily 1 group d member 1 (Nr1d1), metastasis associated lung adenocarcinoma transcript 1 (Malat1), and forkhead box a1 (Foxa1) genes was significantly upregulated in *Hmox1* deficient cells (Figure 6). There were no changes in E2f transcription factor 1 (E2f1), eukaryotic translation initiation factor 1 (Eif1), heterogeneous nuclear ribonucleoprotein a2/b11 (Hnrnpa2b), or ubiquitin a-52 residue ribosomal protein fusion product 1 (Uba52) genes between *Hmox1* deficient and WT cells (Figure 6). This suggests that HO-1 modulates gene expression in a gene-specific manner, and not all genes that are predicted to bind to HO-1 protein are transcriptionally regulated under these conditions.

### 3.9. Mutation of Amino Acids in HO-1 DBD1 Alters Target Gene Expression

**Figure 7:**
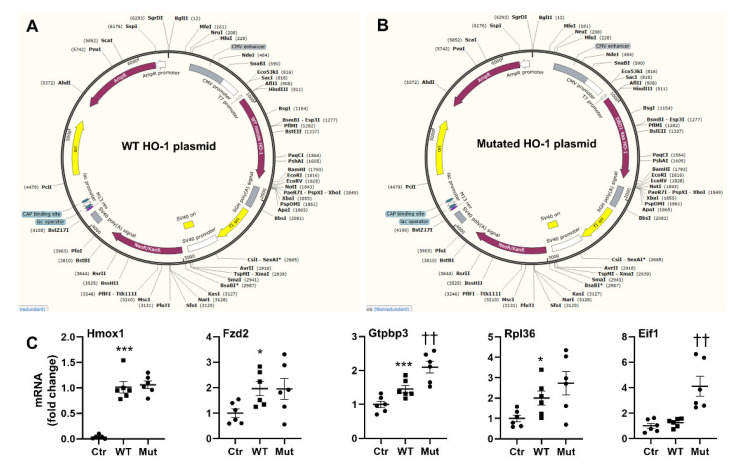
Mutation of amino acids in HO-1 DBD1 alters target gene expression. (**A**,**B**) Construction of WT and mutated HO-1 plasmids. In mutated plasmids, four conserved amino acids (E215, I211, E201, and Q27) within the HO-1 DBD1 were mutated to alanine. (**C**) Gene expression was measured by qRT-PCR in *Hmox1* deficient MLE-12 cells transfected with WT and mutated (Mut) HO-1 plasmids, or control without plasmids (Ctr). Data are expressed as mean ± SEM. N = 6. * *p* < 0.05, *** *p* < 0.001 vs. Ctr group, ^††^ *p* < 0.01 vs. WT group.

To further validate HO-1 as a DNA binding protein, we constructed plasmids of wild type and mutated HO-1 (Figure 7A,B). In mutated plasmids, four amino acids (E215, I211, E201, and Q27) within the HO-1 DBD1 were mutated to alanine. These were chosen since they are conserved between mouse and human. We then transfected these plasmids into *Hmox1* deficient MLE-12 cells and measured expression of Fzd2, Gtpbp3, Rpl36, and Eif1 genes. We chose these genes, as they were identified within the top 10 binding hits normalized by gene length predicted to bind DBD-1, but are not in the top 10 binding hits for DBD-2 or DBD-3. As shown in Figure 7C, mutation of these amino acids within the HO-1 DBD-1 did not affect *Hmox1* gene expression, but significantly increased the expression of Gtpbp3 and Eif1 genes. These results suggest that amino acids E215, I211, E201, and Q27 within the DBD1 play important roles in binding and regulating expression of target genes.

## 4. Discussion

Previous studies have shown that HO-1 protein can enter the nucleus and bind to nuclear proteins, which regulate transcriptional factors including AP1, AP2, Brn3, Stat1, Stat3, and Stat4 [11,12,14,39]. In addition, the DNA binding activity of NF-κB and stimulatory protein (SP)-1 are repressed when both nuclear and cytoplasmic HO-1 protein is overexpressed 21. In addition, during oxidative stress, nuclear HO-1 binds to Nrf2 to prevent its degradation, thereby prolonging its action [40]. It is unclear whether HO-1 can directly bind to DNA and modulate gene expression. In the present study, using Chip-seq, we demonstrated, for the first time, that HO-1 is a putative DNA binding protein and identified three probable HO-1 DNA binding domains using 3D structural modeling. By using protein–protein interaction networks, we identified Uba52, Ubc, Hist2h2ab, Hist2h2ac, Ddx5, Eef1a1, Rpl3, Rpl9, Rpl27, Rpl36, and Hnrnpa3 as the most highly connected nodes in the interactome among the HO-1 gene binding targets identified by CHIP-seq. We validated the impact of HO-1 mutations within the DBD-1 and disruption on the expression of the target genes. Therefore, HO-1 protein putatively binds to DNA, which may regulate target gene expression. The expression of HO-1 is low under normal physiological conditions. Further studies are warranted to investigate the effect of HO-1 on target genes under oxidative stress.

Unlike a DNA electrophoretic mobility shift assay, ChIP-seq allows for the characterization of a protein that binds to DNA as well as the sequences of the oligos from the immunoprecipitated protein-DNA complex [41]. Using this technology, we showed that HO-1 bound to specific DNA sequences. Future studies using surface plasmon resonance and isothermal titration calorimetry as complementary methods would further validate the interaction of HO-1 with DNA. One third of the DNA hits were within promoter regions, suggesting that this could alter transcriptional regulation. In addition, another third of the binding sites found within distal intergenic regions could still have an effect on gene transcription and processing. Transcription factors bind to enhancer or promoter regions to cause an effect on target gene transcription. It is unclear whether HO-1 interacts directly with transcription factors, the spliceosome, coactivators or corepressors, which co-modulates targeted gene expression. It will also be useful to verify our findings in other cell types to determine whether the binding of HO-1 protein to DNA is ubiquitous and not cell-specific. A luciferase reporter assay is warranted to establish a functional connection between the presence of HO-1 protein and the amount of gene product synthesized.

The murine apo HO-1 protein structure is not available in the Protein Data Bank. Many domains of HO-1 protein, including the alpha helical fold, are highly conserved among bacteria, mice, and humans [16,42,43]. Thus, we used the human apo HO-1 structure for modeling with ModelX and FoldX software. The murine structure would have been preferred since the ChIP-Seq was done in murine cells. We identified three putative novel DNA binding domains on human apo HO-1 protein with nucleotide resolution of specific compatible double-stranded DNA (dsDNA) binding sequences. Crystallography could conclusively demonstrate the co-crystallization of the DNA targets found here.

Using the refined nucleotide sequences for each of the unique dsDNA fragments predicted to bind the three predicted DBDs, we were able to search the genes found to bind HO-1 by ChIP-Seq for compatible binding sites within each gene. We showed that most genes found to bind HO-1 by ChIP-Seq had at least one predicted HO-1 compatible binding sequence and identified genes which had the highest proportion of HO-1 compatible binding sequences normalized by their gene length. The most energetically favorable binding sequences for all three sites had a high proportion of GC content, which suggests that HO-1 DNA binding favors GC rich regions such as gene regulatory domains. These GC-rich sequences are often located in gene promoters and play a role in transcription initiation. For instance, 76% of human promoters are GC-rich and contain multiple binding sites of the transcriptional activator SP1 [44]. Whether these GC-rich regions are located in the promoters of HO-1 targeted genes remains unknown.

We observed that HO-1 DBD-2 overlaps with the heme-binding functional ring of HO-1 on peptide residue His25. It is possible that HO-1 His25 is not available for binding heme when dsDNA is bound to DBD-2. This could explain the loss of HO-1 enzymatic activity when HO-1 enters the nucleus. It remains unclear whether heme is competing for the binding of HO-1 to DNA, although it is possible since heme has been found in small quantities within the nucleus [45]. DBD-1 contains Gln27 on chain B, but the heme binding domain is on chain A (opposite side of the protein). However, DBD-2 is on chain A and its peptides surround the heme binding functional ring on His25. Mutation of His25 significantly reduces HO-1 activity [46]. It remains unclear whether DBD-1 and DBD-2 domains are essential for HO-1 protein folding or enzymatic activity. This could be validated using our HO-1 DBD-1 mutated cells.

HO-1 can undergo post-translational modifications (PTMs), including phosphorylation and acetylation, which affects its localization and function [14,47,48,49]. We observed that DNA binding domains contain or are very proximal to amino acids known to have PTMs in HO-1. This may affect or allow binding of HO-1 protein to targeted genes. For instance, DBD-1 is close to Tyr114 which can be phosphorylated and to Arg196 which can be acetylated. DBD-1 also contains the binding site of a known HO-1 inhibitor QC-308 [50]. Whether QC-308 causes inhibition of HO-1 by preventing HO-1 protein-DNA binding remains to be explored. While DBD-2 is likely to be regulated by ubiquitination at Lys22, other Lys residues including Lys18 and Lys 20 may promote ubiquitination or acetylation of HO-1 protein. Nevertheless, many residues in the DBDs have no PTMs [51]. Further study is required to determine whether mutation of these and other residues affects the binding of HO-1 protein to DNA. Importantly, screening drugs that only affect the DNA-binding function of HO-1 is an exciting future direction and may have important implications for health.

Of the gene targets explored, HO-1 deficiency caused transcriptional changes on Dnajb1, Eef1a1, Rpl27, Ubc, Hmgb2, Malat1, Foxa1, and Nr1d1. This suggests that HO-1 may at least partially regulate these genes through DNA transcription. These genes are involved in DNA flexibility, translation initiation, protein folding, differentiation, proliferation, and circadian oscillation [37,52,53,54,55], corroborating the multiple functions of HO-1. Nr1d1 is also a target gene of Nrf2 [56]. Further studies are required to determine whether HO-1 interacts with other transcription factors including Nrf2 which regulates expression of these genes. A previous study comparing *Hmox1* deficient and WT induced pluripotent stem cells reported differential gene expression of over 15 enriched biological process GO Terms [57]. Our study showed congruence of six biological process terms. This includes proliferation, differentiation, embryogenesis, DNA repair and tumorigenesis. These results can be used as a guide for further investigation of these potential targets.

## 5. Conclusions

HO-1 protein preferentially binds to DNA at specific DNA binding sequences or motifs within three novel putative binding sites, including one within its enzymatic pocket. This strongly suggests that the function of HO-1 is not only enzymatic, and regulatory through protein–protein binding, but also regulatory through DNA binding and transcriptional regulation. Thus, our study provides the first characterization of HO-1 as a putative DNA-binding protein and begins the foundation for developing specific inhibitors or activators targeting HO-1 DNA binding domains to modulate targeted gene expression and corresponding cellular function.

## Figures and Tables

**Table 1:** Top 10% of genes with highest *p*-value probability of DNA binding to HO-1 and known literature interactions to HO-1.

Genes	Description = Known Connection to HO-1	Relationship to HO-1
Bag3	Mus musculus BCL2-associated athanogene 3 (Bag3), mRNA.	In HO-1 gene ontology maps, Hmox1->Hsf1->Bag3
Coq9	Mus musculus coenzyme Q9 (Coq9), mRNA.	Less ox stress = less Coq9
Dnajb1	Mus musculus DnaJ heat shock protein family (Hsp40) member B1 (Dajb1), transcript variant 1, mRNA.	Mediated by Nrf2
Dpp10	Mus musculus dipeptidylpeptidase 10 (Dpp10), mRNA. (Asthma)	Related to Asthma
Eef1a1	Mus musculus eukaryotic translation elongation factor 1 alpha 1 (Eef1a1), mRNA.	HO-1-interacting-protein
Fzd2	Mus musculus frizzled class receptor 2 (Fzd2), mRNA.	Inhibited by cigarette smoke
Hist1h1e	Mus musculus histone cluster 1, H1e (Hist1h1e), mRNA.	Induced by oxidation
Hmgb2	Mus musculus high mobility group box 2 (Hmgb2), mRNA.	Hmbg1 inhibited by HO-1
Hnrnpa2b1	Mus musculus heterogeneous nuclear ribonucleoprotein A2/B1 (Hnrnpa2b1), transcript variant 1, mRNA.	Mediated by Nrf2
Lias	LIAS (Lipoic Acid Synthetase)	Connected to Coq9 and Bola3 Iron Sulfur metabolism
Mat2a	Mus musculus methionine adenosyltransferase II, alpha (Mat2a), mRNA.	Negative correlation with HO-1
Opa1	Mus musculus OPA1, mitochondrial dynamin like GTPase (Opa1), transcript variant 2, mRNA.	PKC-α/HO-1 induced
Rn7sk	Mus musculus RNA, 7SK, nuclear (Rn7sk), small nuclear RNA.	Inhibited by HO-1

**Table 2:** Summary of dsDNA-peptide contacts for three HO-1 DNA binding domains.

HO-1 DNA Binding Domains	DBD-1	DBD-2	DBD-3
Predicted binding dsDNA fragment length (number of nucleotides)	10	10	10
Protein residue contacts	9	7	8
DNA contacts	7	6	5

Here, we report the fragment length (in number of nucleotides) of each predicted dsDNA binding fragment predicted by ModelX, the number of HO-1 protein residues contacting the dsDNA fragments, and the number of DNA fragment positions which contact with protein residues for each of the newly described three HO-1 DNA binding domains.

**Table 3:** Specific dsDNA-peptide contact positions within each HO-1 DNA binding domain.

HO-1 DBD-1								
Non-specific contacts between DNA strand and protein backbone								
DNA	gA6	aB20						
Protein	QB197	RB198	IB211					
Non-specific contacts between DNA strand and protein side chains								
DNA_SC	gA6	gA12	tA13	cB19	aB20	tB21	gB22	
Protein_SC	RA100	QB27	RB113	QB197	EB201	IB211	QB212	EB215
HO-1 DBD-2								
Contacts between DNA strand and protein backbone								
DNA	tB29	cB30						
Protein	PA10	AA20						
Contacts between DNA strand and protein side chains								
DNA_SC	gA8	aA9	gB27	aB28	tB29	cB30		
Protein_SC	PA10	AA16	AA20	KA22	TA26	QA197	IA200	
HO-1 DBD-3								
Contacts between DNA strand and protein side chains								
DNA_SC	cA18	aA19	tA20	gA21	tB38			
Protein_SC	KA116	RA117	EA120	EA125	RA198	EA202	KA204	LA208

Here, we report the specific protein residues and nucleotides which make contact in the HO-1 DNA binding complex in each of the three HO-1 DNA Binding Domains. DNA base descriptions follow the format: nucleotide, strand (A or B), and ModelX assigned fragment base number (Strand A bases: 1–10 forward-reverse/Strand B bases: 11–20 reverse-forward). Protein residue descriptions follow the format: one letter amino acid code, protein chain, and protein residue number. Protein residues and DNA base positions match the HO-1 DNA Binding Complex Structure PBD files provides in the Supplementary Materials.

## Data Availability

All of the original data for the article are available upon reasonable request.

## Supplementary Materials

The following are available online at https://www.mdpi.com/article/10.3390/antiox11112135/s1, Supplementary Figure S1. Kmer content plots. Supplementary Figure S2. Read quality plots. Supplementary Figure S3. GC content plots. Supplementary Figure S4. GO term (biological processes) bubble plots. Supplementary Figure S5: Full-length blot of Figure S6A. Supplementary Table S1. A second QC analyses of trimmed reads was performed in FastQC (V0.11.5). Supplementary Table S2. Initial QC analyses of raw reads was performed in FastQC (V0.11.5). Supplementary Table S3. Kmer content table—GroupA_HO-1_1 Trimmed reads. Supplementary Table S4. Alignment summary statistics. Supplementary Table S5. WGS summary statistics. Supplementary Table S6. Duplication statistics. Supplementary Table S7. Gene list observed in the HO-1 ChIP experiment.
