# Supplementary material for: Identification of Heme Oxygenase-1 as a Putative DNA-Binding Protein
Source: Antioxidants (Basel). 2022 Oct 28;11(11):2135. doi: 10.3390/antiox11112135 (PMC9686683; doi:10.3390/antiox11112135)
Supplement: Supplementary file 1 [file antioxidants-11-02135-s001.zip › antioxidants-1860249-supplementary.pdf]

Supplementary materials

Identification of heme oxygenase-1 as a putative DNA-binding protein

Alejandro Scaffa[1†], George A. Tollefson[2*†], Hongwei Yao[1*†], Salu Rizal[1], Joselynn Wallace[3], Nathalie Oulhen[1], Jennifer Carr[1], Katy Hegarty[1], Alper Uzun[4,5,6], Phyllis A. Dennery[1,4]

[1]Department of Molecular Biology, Cell Biology & Biochemistry, Division of Biology and Medicine, Brown University, Providence, RI 02912, USA; [2]Department of Pathology and Laboratory Medicine, Rhode Island Hospital, Providence, RI 02903, USA; [3]Center for Computational Biology of Human Disease, and Center for Computation and Visualization, Brown University, Providence, RI 02906, USA; [4]Department of Pediatrics, Warren Alpert Medical School of Brown University, Providence, RI 02903, USA; [5]Department of Pediatrics, Women and Infants Hospital, Providence, RI 02905, USA; [6]Center for Computational Molecular Biology, Brown University, Providence, RI 02906, USA.

To whom correspondence should be addressed:
Phyllis A. Dennery, M.D.,
Departments of Pediatrics and
Cell Biology, Molecular Biology and Biochemistry
Warren Alpert Medical School of Brown University
593 Eddy St Suite 125
Providence RI 02903
Phone: 401-444-5648
Email: phyllis_dennery@brown.edu

[†]These authors contributed equally to this work.

Supplementary Figure S1. Kmer content plots. The x-axis represents the position along the length of the read, and the y-axis represents the relative enrichment of each kmer. The untrimmed reads are shown on the left, and trimmed reads are shown on the right.

Library	Raw reads	Trimmed reads
GroupA_LH20_1	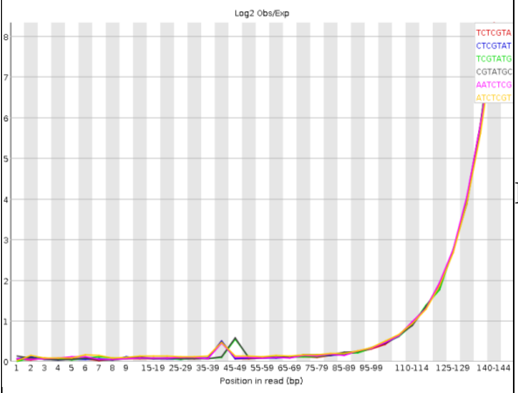	No enriched Kmers
GroupA_LH20_2	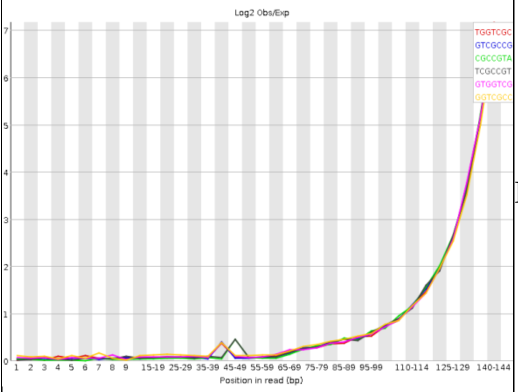	No enriched Kmers
GroupA_IgG_1	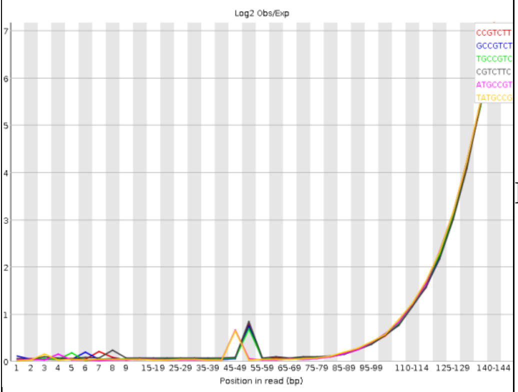	No enriched Kmers

GroupA_IgG_2	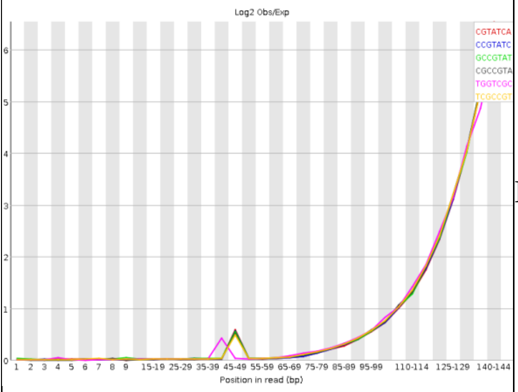	No enriched Kmers
GroupA_HO-1_1	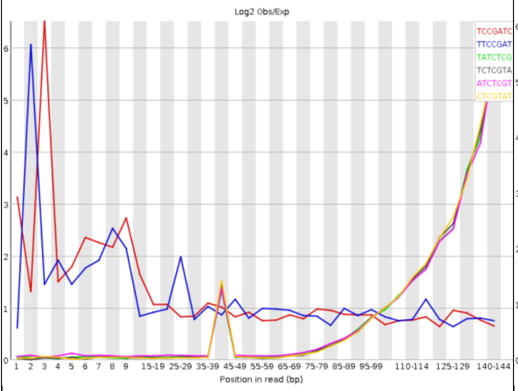	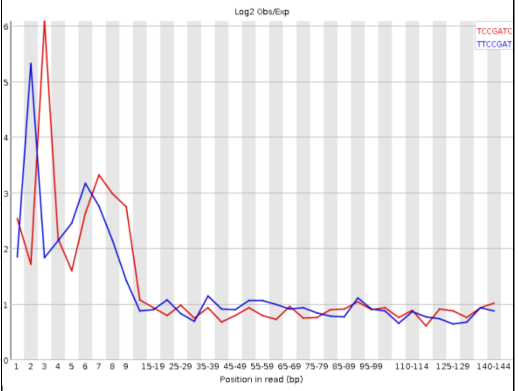
GroupA_HO-1_2	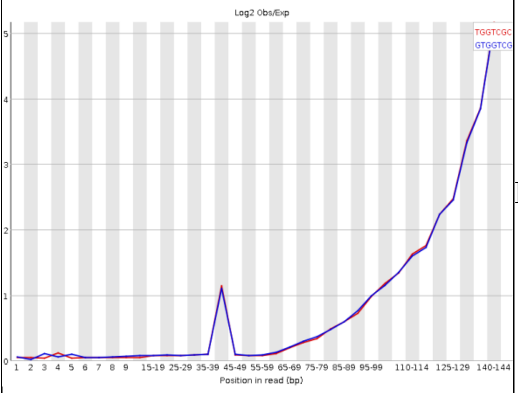	No enriched Kmers

GroupB_H20_1	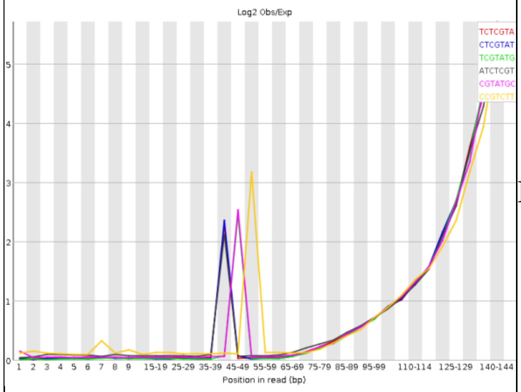	No enriched Kmers
GroupB_H20_2	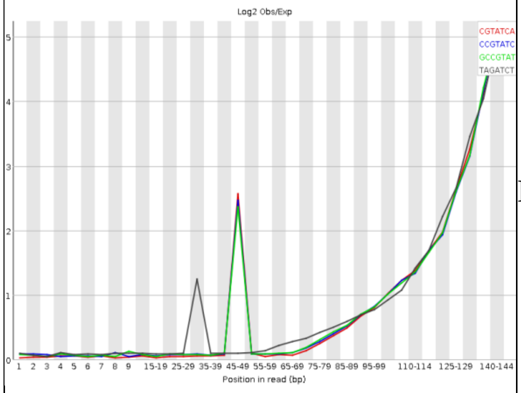	No enriched Kmers
GroupB_IgG_1	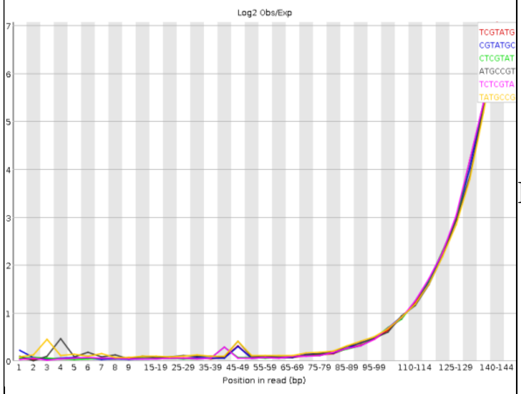	No enriched Kmers

<table>
<tr><td>GroupB_IgG_2</td><td>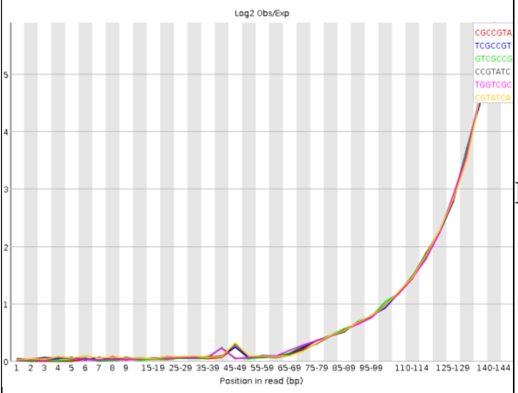
</td><td>No enriched Kmers</td></tr>
<tr><td>GroupB_HO-1_1</td><td>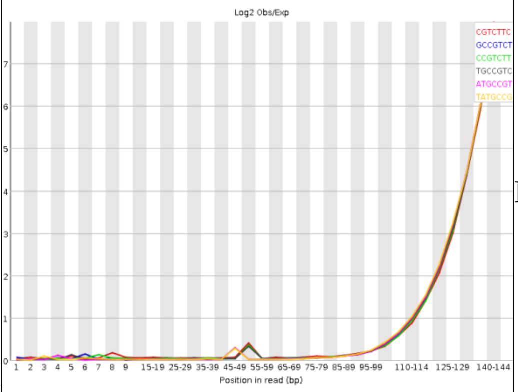
</td><td>No enriched Kmers</td></tr>
<tr><td>GroupB_HO-1_2</td><td>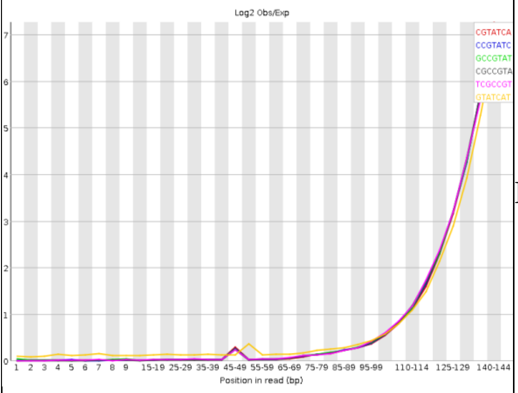
</td><td>No enriched Kmers</td></tr>
</table>

Supplementary Figure S2. Read quality plots. The x-axis represents the position along the length of the read, and the y-axis represents the quality scores. The yellow box represents the interquartile range of the quality score, the red line is the median, the blue line is the mean, and the whiskers are the 10th and 90th percentiles. The untrimmed reads are shown on the left, and trimmed reads are shown on the right.

Library	Raw Reads	Trimmed Reads
GroupA_LH20_1	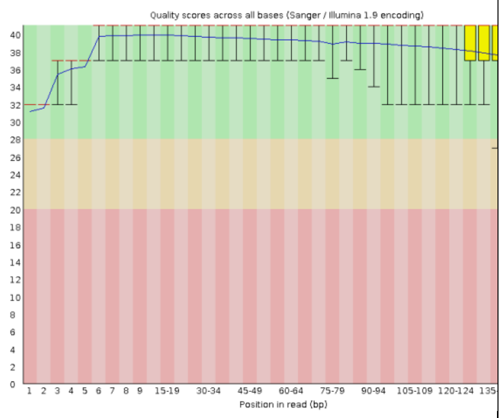	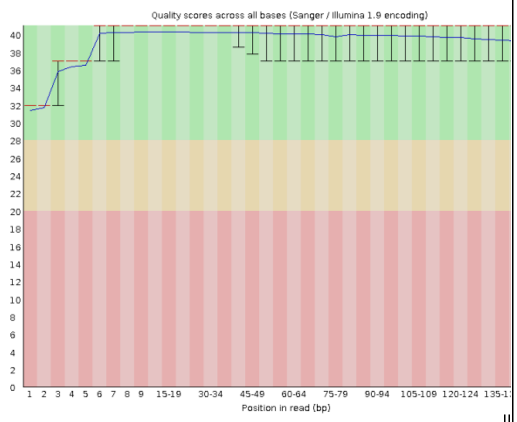
GroupA_LH20_2	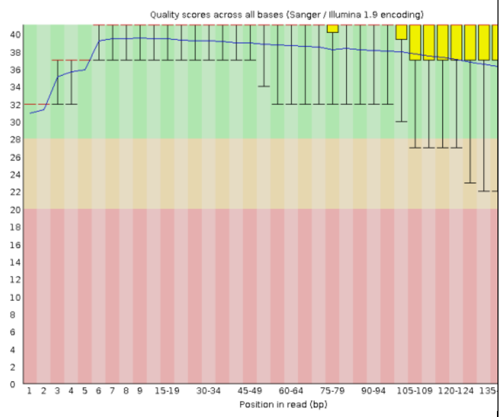	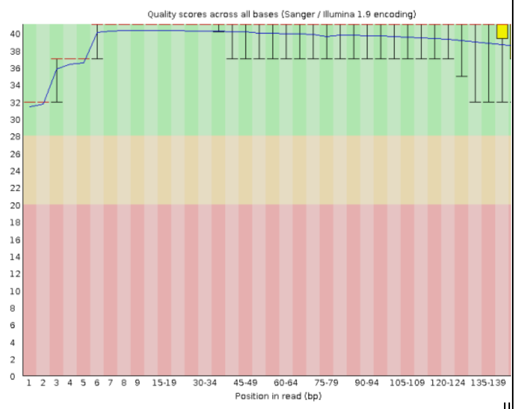

GroupA_IgG_1	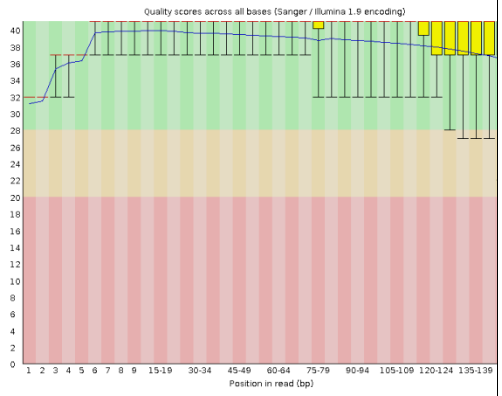	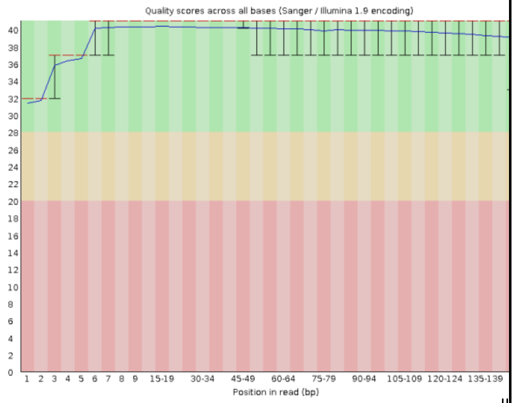
GroupA_IgG_2	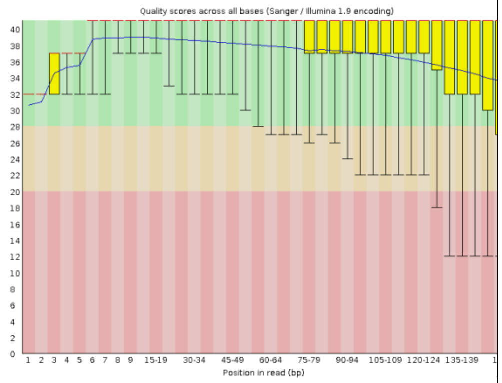	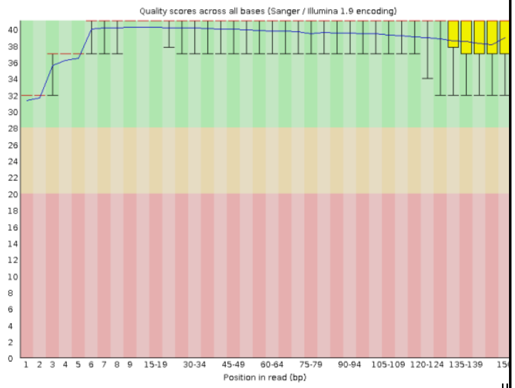
GroupA_HO-1_1	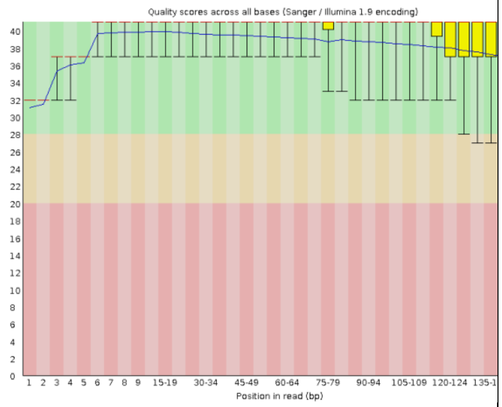	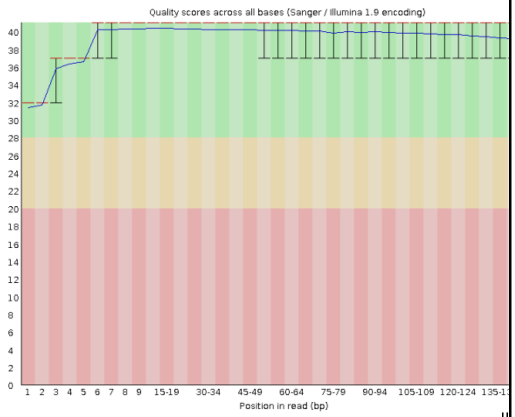

GroupA_HO-1_2	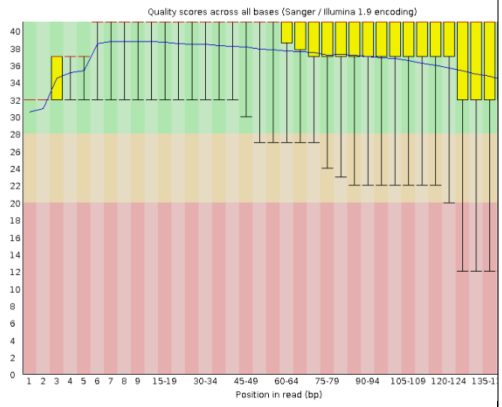	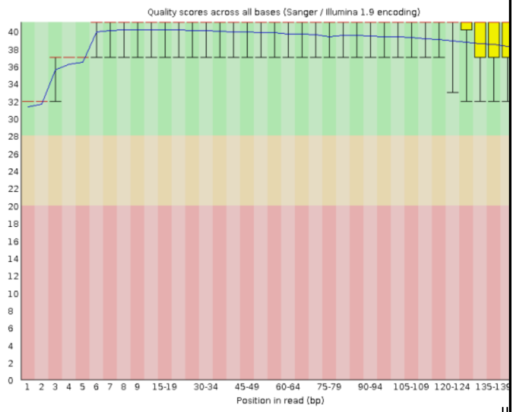
GroupB_H20_1	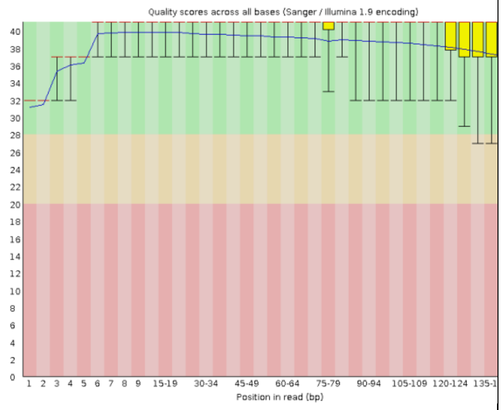	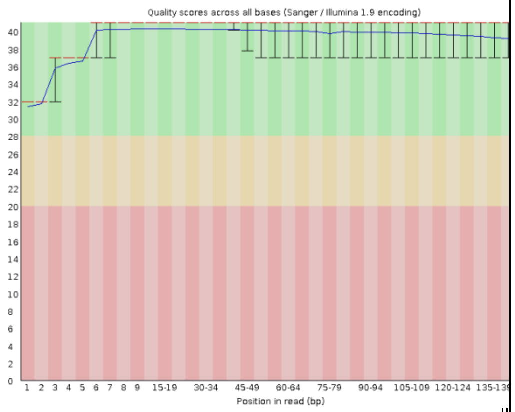
GroupB_H20_2	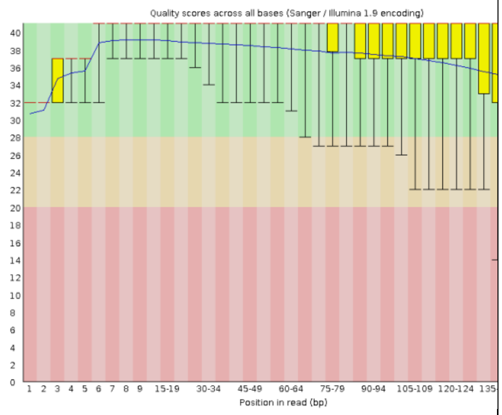	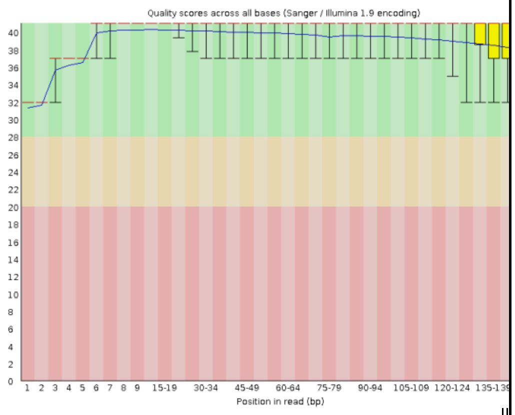

GroupB_IgG_1	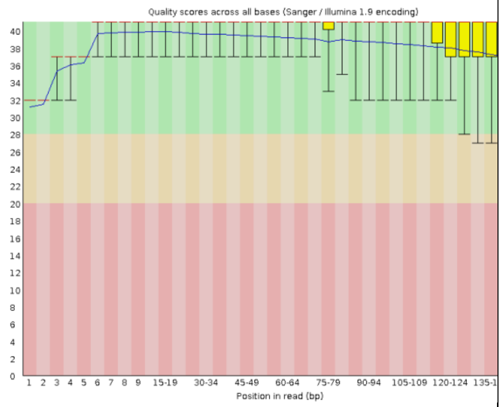	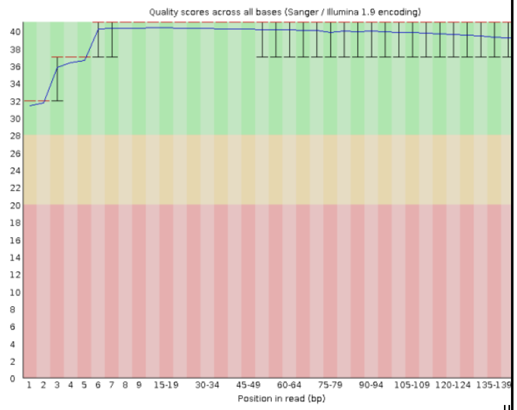
GroupB_IgG_2	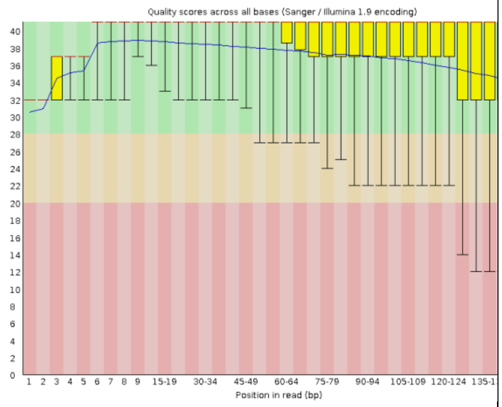	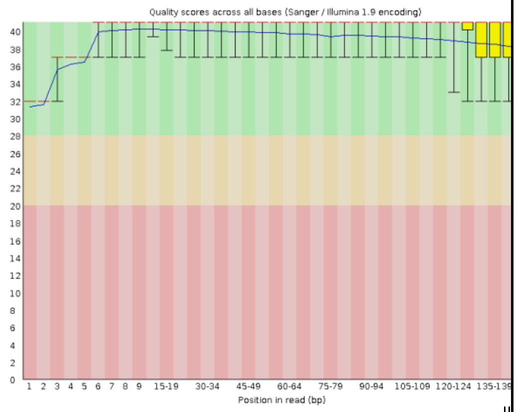
GroupB_HO-1_1	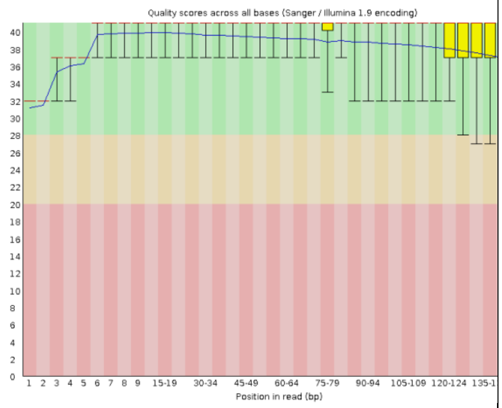	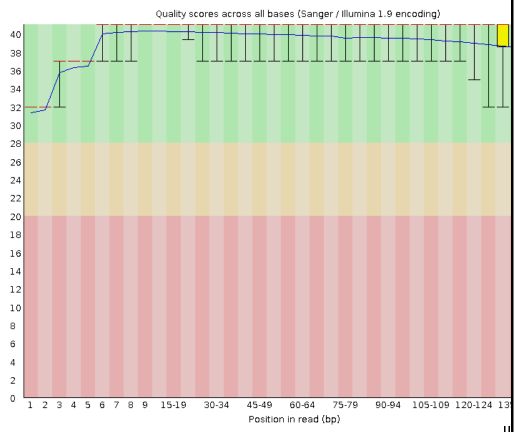

GroupB_HO-1_2	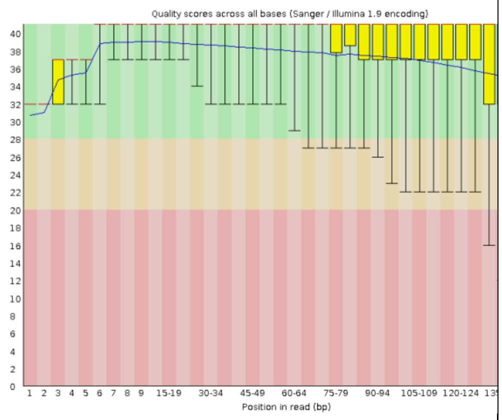Quality scores across all bases (Sanger / Illumina 1.9 encoding)<br>Position in read (bp)	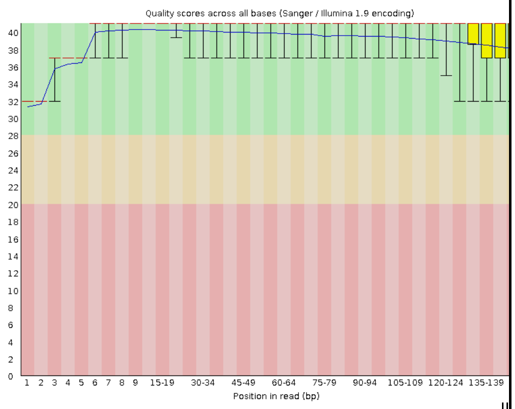Quality scores across all bases (Sanger / Illumina 1.9 encoding)<br>Position in read (bp)

Supplementary Figure S3. GC content plots. The x-axis represents percent GC content and the y-axis shows the number of reads with a given GC content. The red line is the observed GC content for a set of reads, and the blue line is a theoretical distribution assuming uniform GC content for all reads. The untrimmed reads are shown on the left, and trimmed reads are shown on the right.

**Library**	**Raw reads**	**Trimmed reads**
GroupA_LH20_1	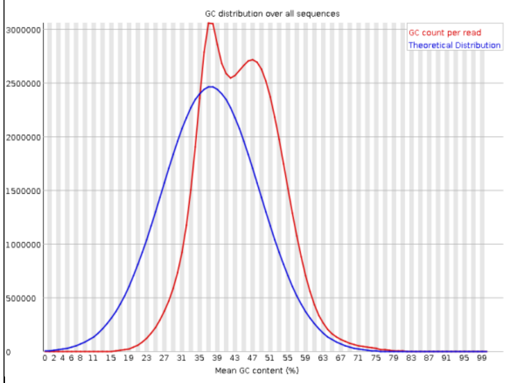	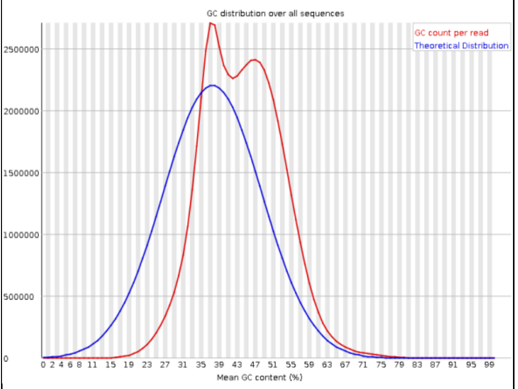
GroupA_LH20_2	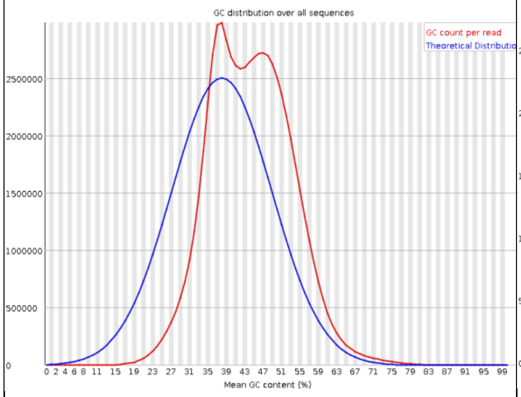	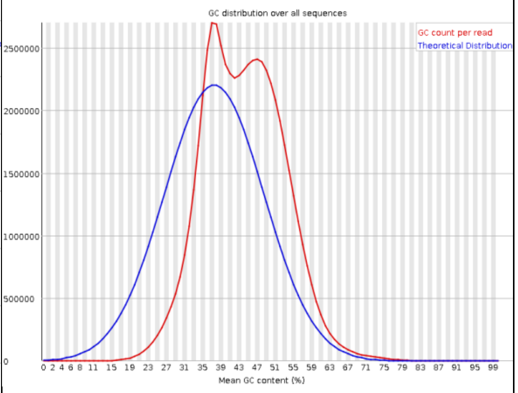
GroupA_IgG_1	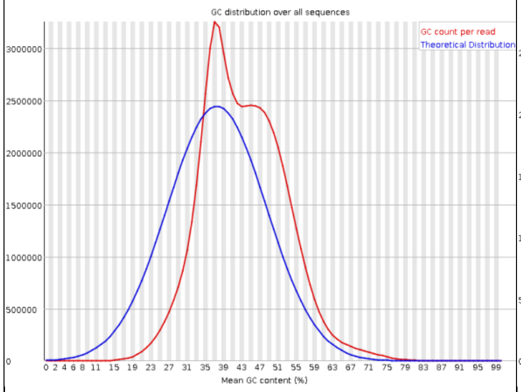	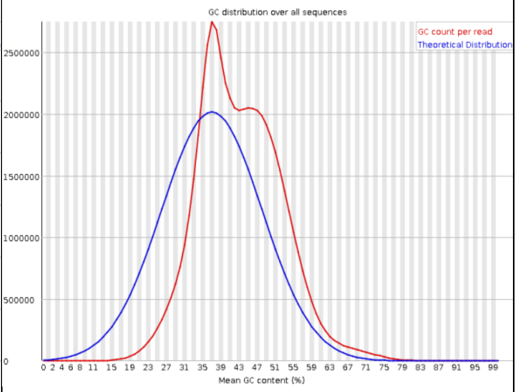

GroupA_IgG_2	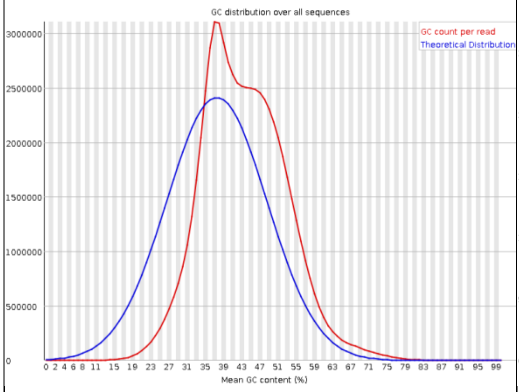	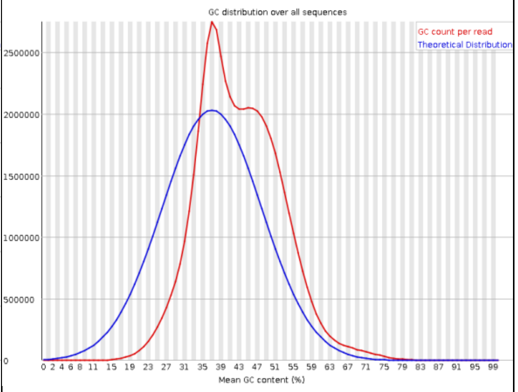
GroupA_HO-1_1	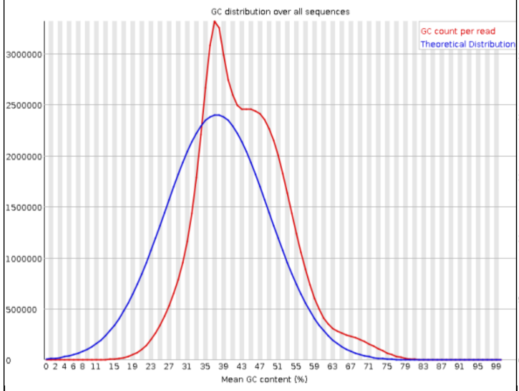	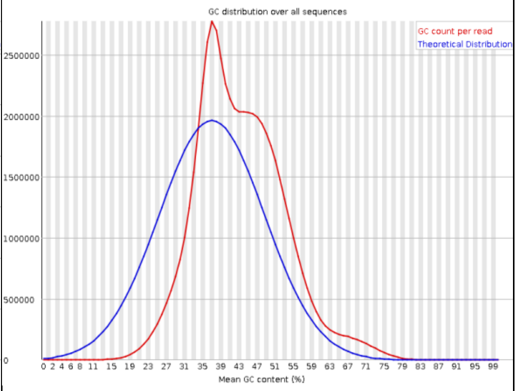
GroupA_HO-1_2	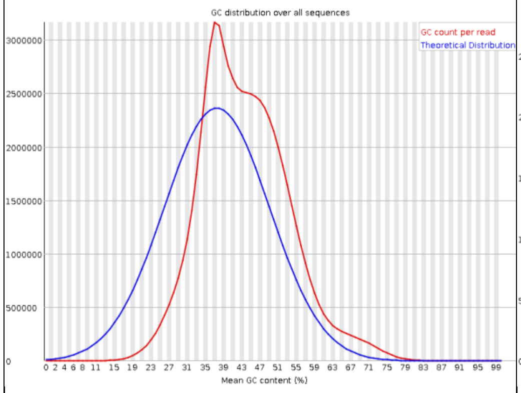	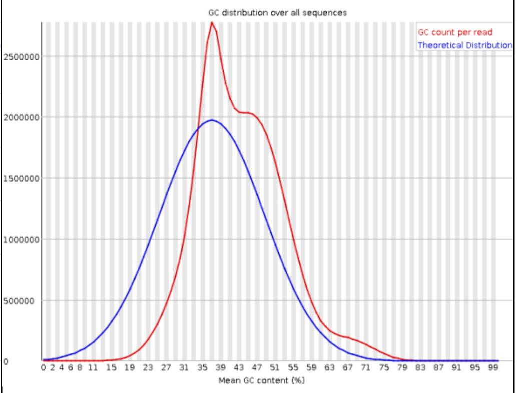

<table>
<tr>
<td>GroupB_H20_1</td>
<td>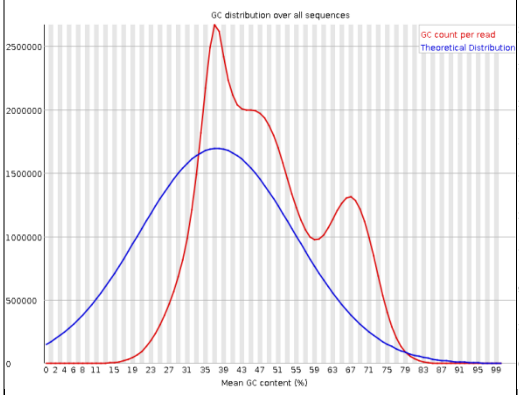
</td>
<td>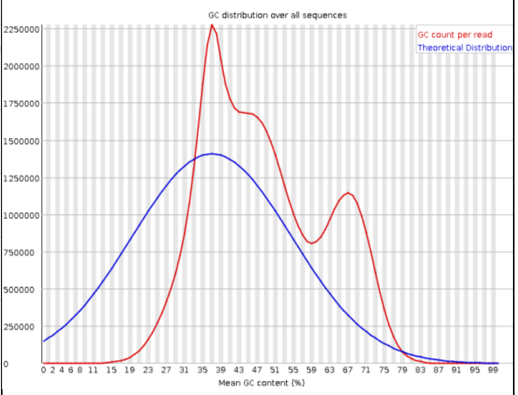
</td>
</tr>
<tr>
<td>GroupB_H20_2</td>
<td>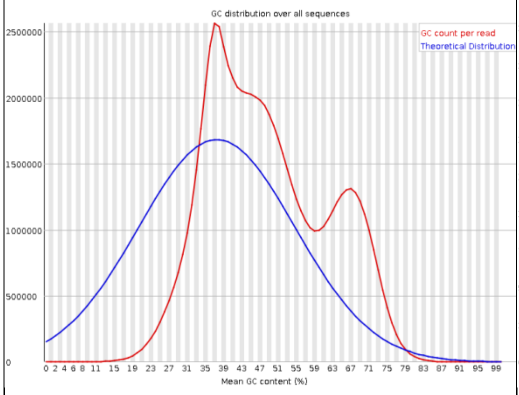
</td>
<td>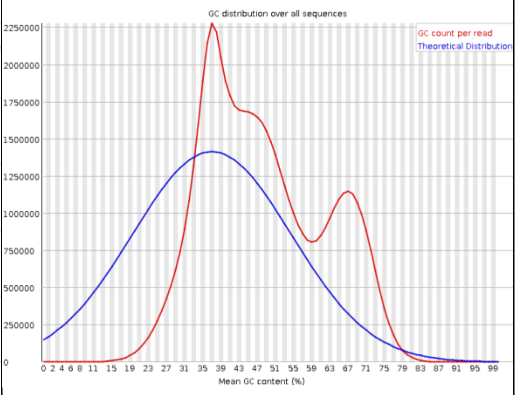
</td>
</tr>
<tr>
<td>GroupB_IgG_1</td>
<td>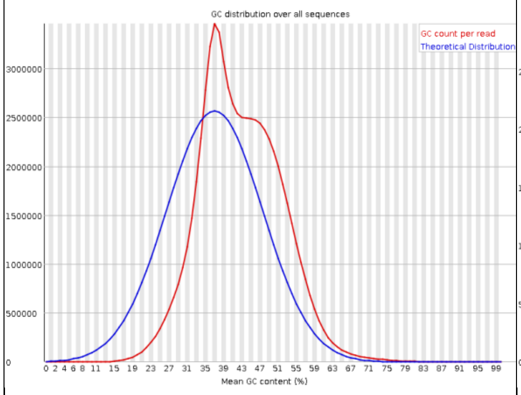
</td>
<td>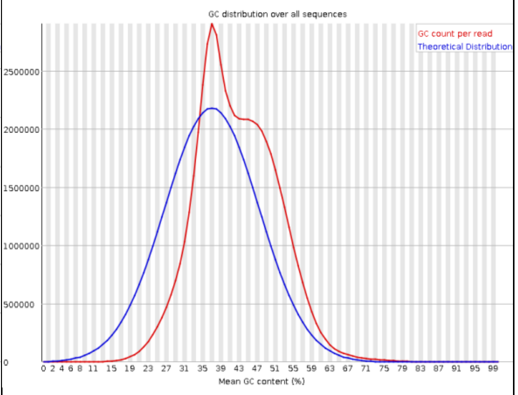
</td>
</tr>
</table>

GroupB_IgG_2	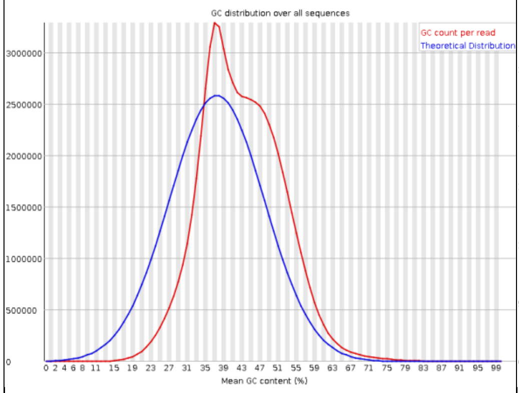	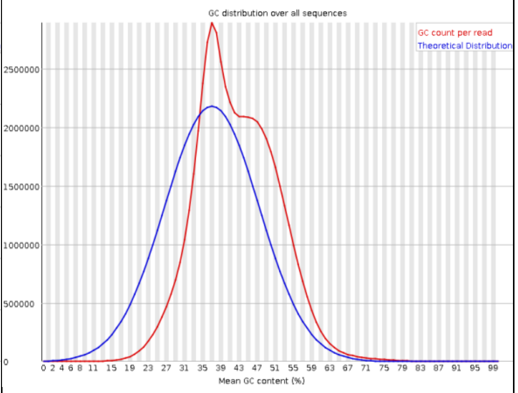
GroupB_HO-1_1	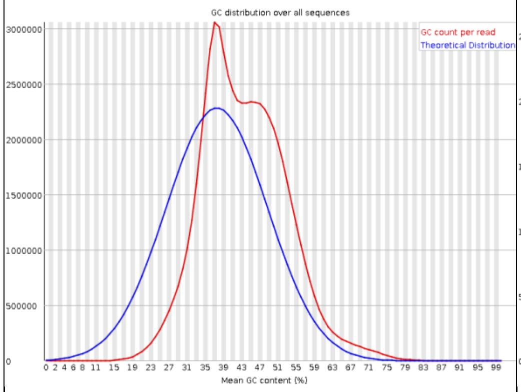	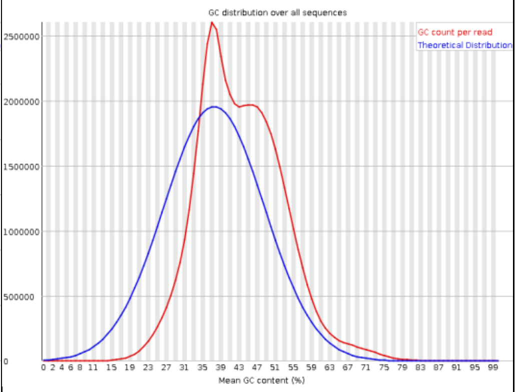
GroupB_HO-1_2	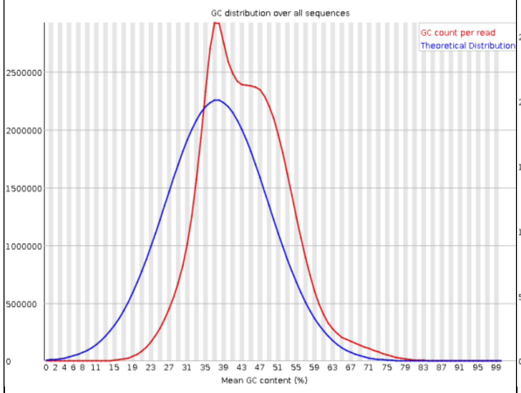	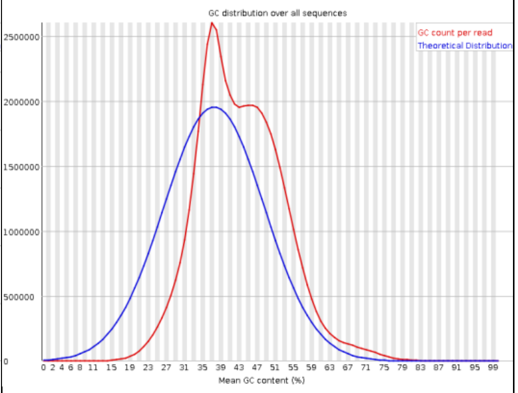

Supplementary Figure S4. GO term (biological processes) bubble plots. Size of the circles represent the number of genes. Red to green color scaling represents the significance of enrichment as -log p value.

A. ChIP-Seq Genes

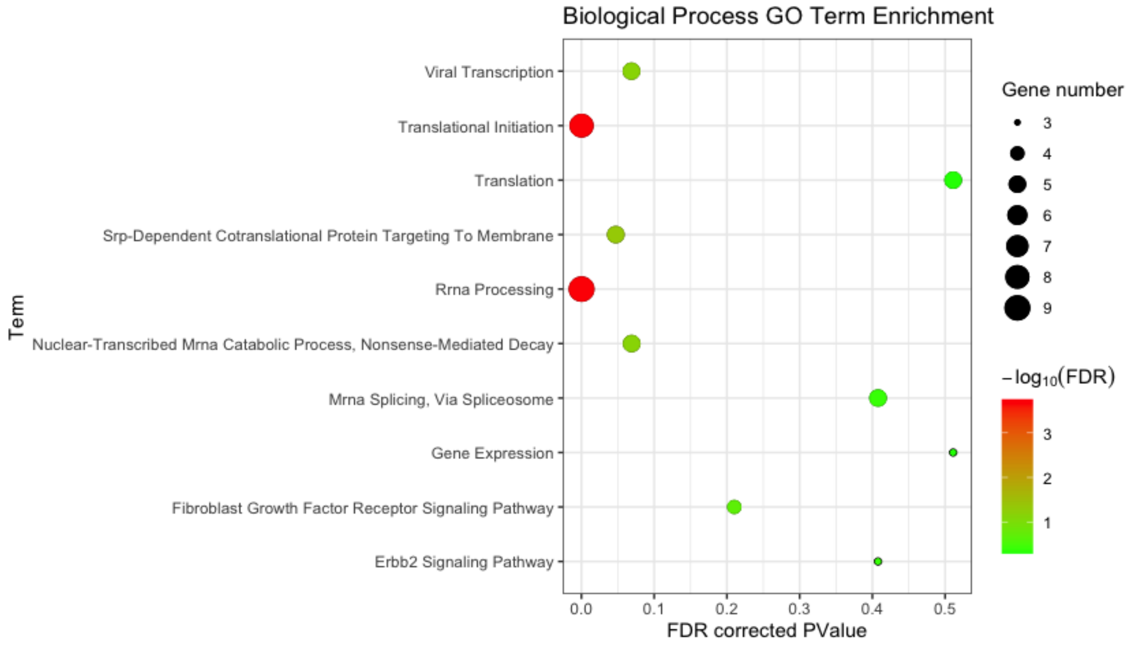

B. HO-1/DBD-1 Top 50% Compatible Binding Genes

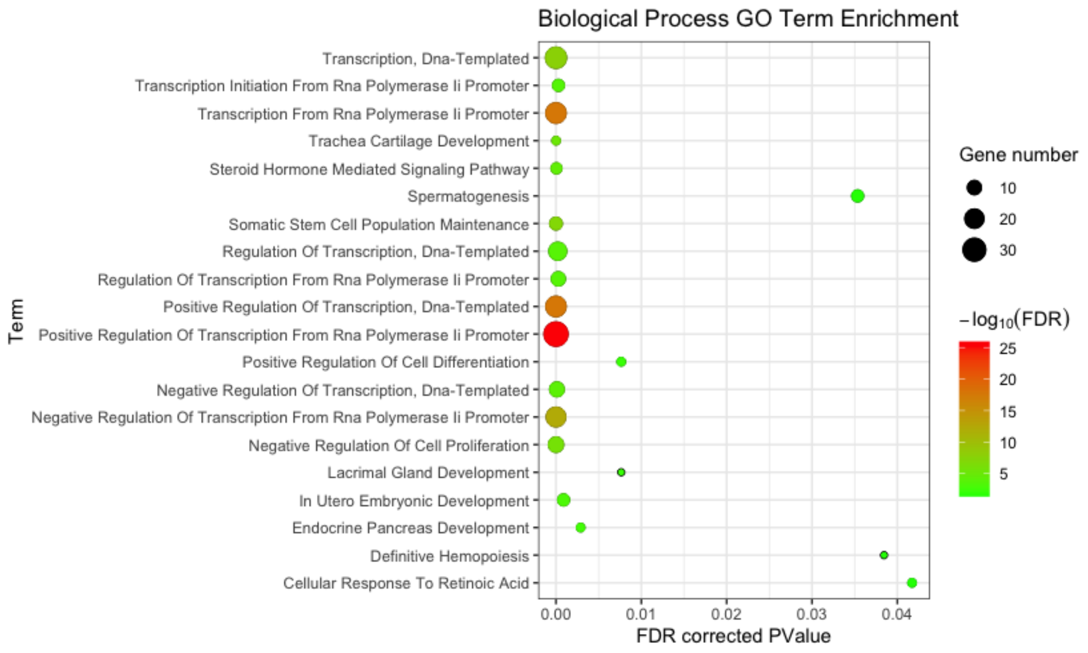

C. HO-1/DBD-2 Top 50% Compatible Binding Genes

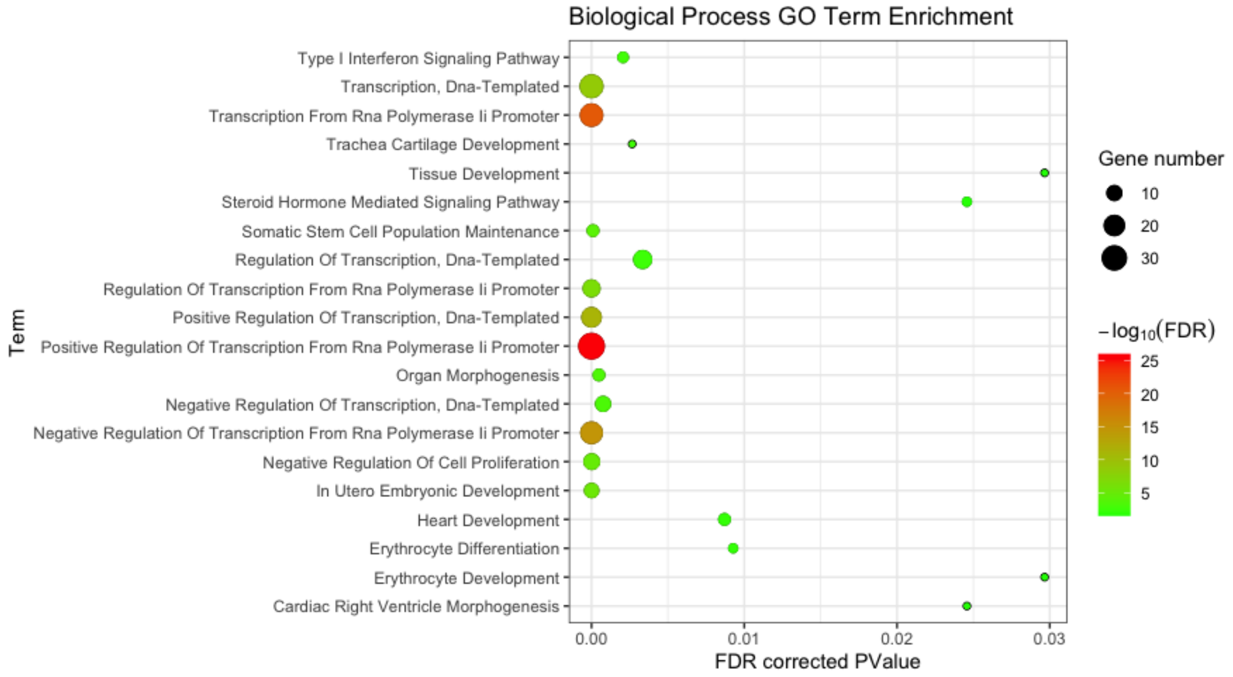

D. HO-1/DBD-3 Top 50% Compatible Binding Genes

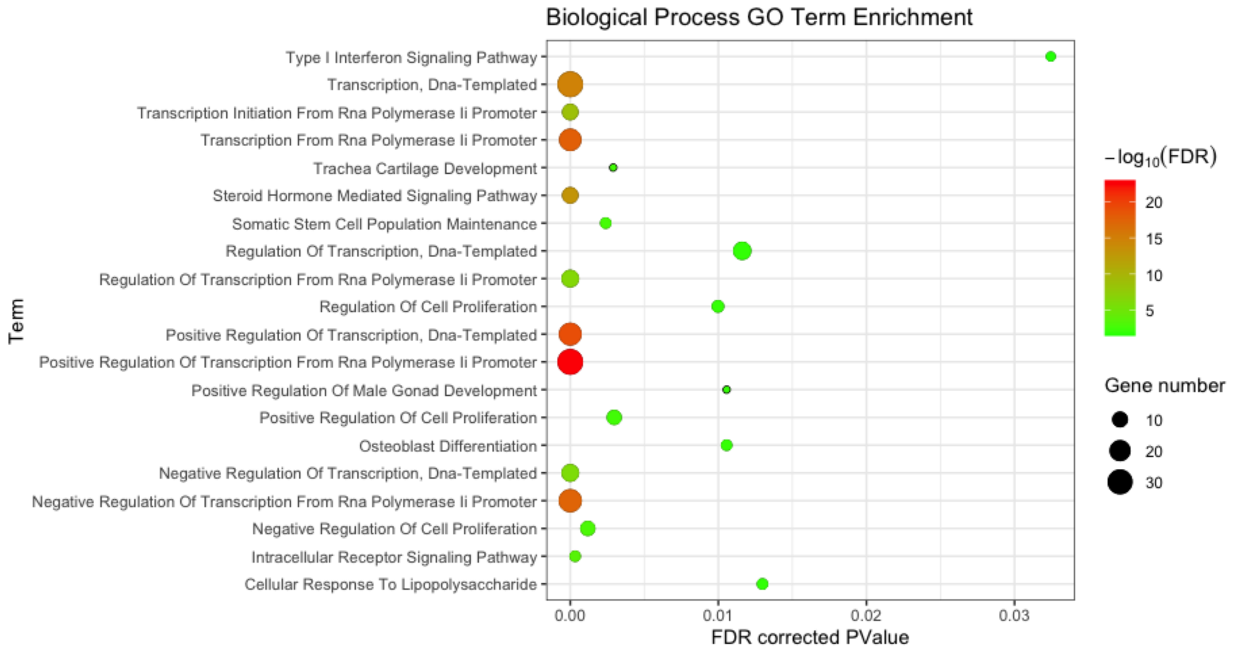

Supplementary Figure S5: Full-length blot of Figure 6A. Representative full Western blot of HO-1 protein in WT and Hmox1 KO (deficient) MLE-12 cells along molecular weight markers. 10 µg of protein was loaded in each lane.

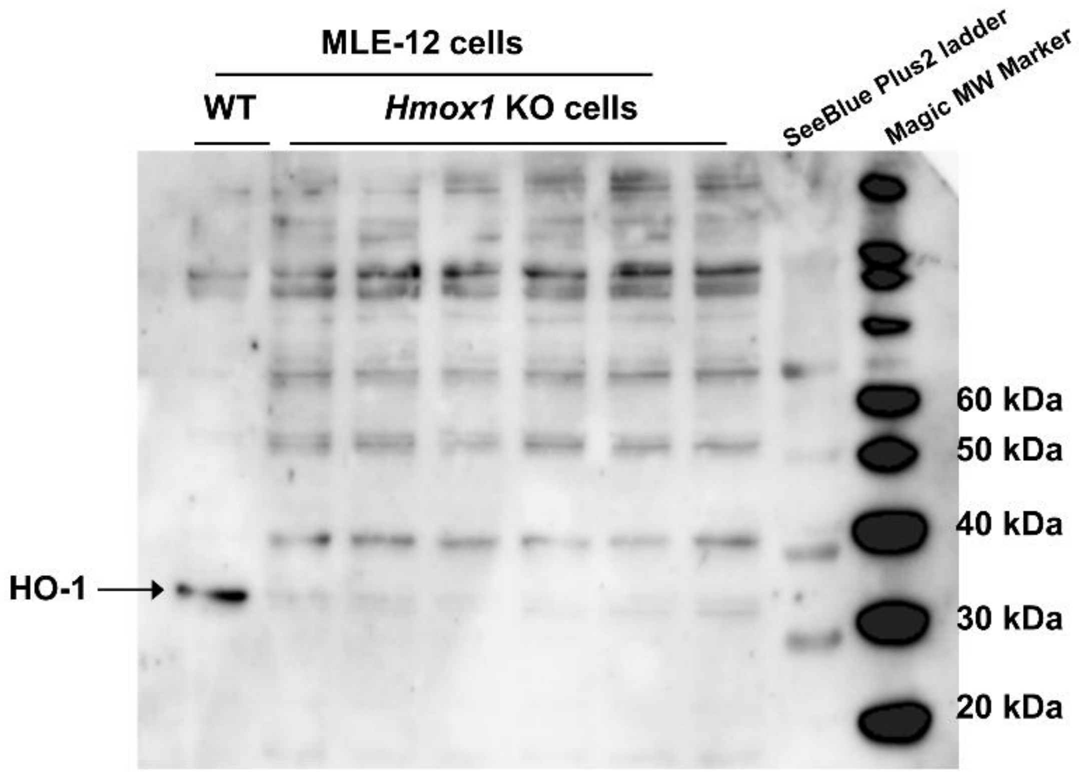

Supplementary Table S1. A second QC analyses of trimmed reads was performed in FastQC (V0.11.5)

Library	# of Raw Reads	Sequence Length	# of poor quality sequences	%GC
GroupA_LH20_1	60123899	75-150	0	43
GroupA_LH20_2	60123899	75-150	0	43
GroupA_IgG_1	55572231	75-150	0	42
GroupA_IgG_2	55572231	75-150	0	42
GroupA_HO-1_1	56866122	75-150	0	42
GroupA_HO-1_2	56866122	75-150	0	42
GroupB_H20_1	61566544	75-150	0	47
GroupB_H20_2	61566544	75-150	0	47
GroupB_IgG_1	56522411	75-150	0	41
GroupB_IgG_2	56522411	75-150	0	41
GroupB_HO-1_1	53668814	75-150	0	42
GroupB_HO-1_2	53668814	75-150	0	42

Supplementary Table S2. Initial QC analyses of raw reads was performed in FastQC (V0.11.5)

Library	# of Raw Reads	Sequence Length	Number of poor quality sequences	%GC
GroupA_LH20_1	68331222	150	0	43
GroupA_LH20_2	68331222	150	0	44
GroupA_IgG_1	66702588	150	0	43
GroupA_IgG_2	66702588	150	0	43
GroupA_HO-1_1	68697828	150	0	43
GroupA_HO-1_2	68697828	150	0	43
GroupB_H20_1	72639136	150	0	48
GroupB_H20_2	72639136	150	0	48
GroupB_IgG_1	67845688	150	0	42
GroupB_IgG_2	67845688	150	0	42
GroupB_HO-1_1	63918020	150	0	43
GroupB_HO-1_2	63918020	150	0	43

Note the slightly higher GC content reported for the GroupB_H20_1 and GroupB_H20_2 libraries relative to the other 10. To remove adapters, low-quality sequences, and short sequences, trimming was performed in Trimmomatic (V0.36) using the following parameters:

Supplementary Table S3. Kmer content table - GroupA_HO-1_1 Trimmed reads

Sequence	Count	p-value	Obs/Exp Max	Max Obs/Exp Position
TCCGATC	6000	0.0	6.081754	3
TTCCGAT	6720	0.0	5.327682	2

Supplementary Table S4. Alignment summary statistics

LIBRARY	TOTAL_READS	READS_ALIGNED_IN_PAIRS
GroupA_LH20	120247798	119552802
GroupA_IgG	111144462	109848730
GroupA_HO-1	113732244	110567302
GroupB_H20	123133088	98219504
GroupB_IgG	113044822	112704592
GroupB_HO-1	107337628	105711984

LIBRARY: The library for which statistics are reported.

TOTAL_READS: The total number of reads including all PF and non-PF reads. When CATEGORY equals PAIR this value will be 2x the number of clusters.

READS_ALIGNED_IN_PAIRS: The number of aligned reads whose mate pair was also aligned to the reference.

Supplementary Table S5. WGS summary statistics

LIBRARY	MEAN_COVERAGE	SD_COVERAGE	PCT_EXC_MAPQ	PCT_EXC_UNPAIRED	PCT_EXC_BASEQ	PCT_EXC_OVERLAP	PCT_EXC_CAPPED	PCT_EXC_TOTAL
GroupA_LH20	3.74	3.42	0.11	1.10E-04	7.14E-03	0.27	0.011	0.40
GroupA_IgG	3.67	3.24	0.12	1.60E-04	7.81E-03	0.20	0.013	0.34
GroupA_HO-1	3.80	3.40	0.11	3.64E-04	8.05E-03	0.20	0.013	0.33
GroupB_H20	2.93	3.46	0.12	7.12E-04	7.55E-03	0.22	0.014	0.36
GroupB_IgG	3.98	3.33	0.11	7.50E-05	8.08E-03	0.18	0.013	0.32
GroupB_HO-1	3.39	3.08	0.11	1.92E-04	7.62E-03	0.22	0.013	0.36

LIBRARY: The library for which statistics are reported.
MEAN_COVERAGE: The mean coverage in bases of the genome territory, after all filters are applied.
SD_COVERAGE: The standard deviation of coverage of the genome after all filters are applied.
MEDIAN_COVERAGE: The median coverage in bases of the genome territory, after all filters are applied.
PCT_EXC_MAPQ: The fraction of aligned bases that were filtered out because they were in reads with low mapping quality (default is < 20).
PCT_EXC_DUPE: The fraction of aligned bases that were filtered out because they were in reads marked as duplicates.
PCT_EXC_UNPAIRED: The fraction of aligned bases that were filtered out because they were in reads without a mapped mate pair.
PCT_EXC_BASEQ: The fraction of aligned bases that were filtered out because they were of low base quality (default is < 20).
PCT_EXC_OVERLAP: The fraction of aligned bases that were filtered out because they were the second observation from an insert with overlapping reads.
PCT_EXC_CAPPED: The fraction of aligned bases that were filtered out because they would have raised coverage above the capped value (default cap = 250x).
PCT_EXC_TOTAL: The total fraction of aligned bases excluded due to all filters.

Supplementary Table S6. Duplication statistics

LIBRARY	READ_PAIRS_EXAMINED	SECONDARY_OR_SUPPLEMENTARY_RDS	UNMAPPED_READS	PERCENT_DUPLICATION	ESTIMATED_LIBRARY_SIZE
Group A_LH20	59776401	681633	659975	0.16	178349158
Group A_IgG	54924365	604280	1264843	0.18	135069209
Group A_HO-1	55283651	649083	3101149	0.22	110761630
Group B_H20	49109752	415352	24781602	0.38	47614157
Group B_IgG	56352296	581808	320129	0.15	172478062
Group B_HO-1	52855992	607151	1592490	0.17	138855851

LIBRARY: The library on which the duplicate marking was performed.
READ_PAIRS_EXAMINED: The number of mapped read pairs examined.
SECONDARY_OR_SUPPLEMENTARY_RDS: The number of reads that were either secondary or supplementary
UNMAPPED_READS: The total number of unmapped reads examined.
PERCENT_DUPLICATION: The fraction of mapped sequence that is marked as duplicate.
ESTIMATED_LIBRARY_SIZE: The estimated number of unique molecules in the library based on PE duplication.

Supplementary Table S7. Gene list observed in the HO-1 ChIP experiment

Gene Name	Description
2410141K09Rik	Mus musculus RIKEN cDNA 2410141K09 gene (2410141K09Rik), transcript variant 2, mRNA.
4930467E23Rik	Mus musculus RIKEN cDNA 4930467E23 gene (4930467E23Rik), mRNA.
4930528G23Rik	Mus musculus RIKEN cDNA 4930528G23 gene (4930528G23Rik), long non-coding RNA.
5330429B09Rik	n/a
Abce1	Mus musculus ATP-binding cassette, sub-family E (OABP), member 1 (Abce1), transcript variant 1, mRNA.
AK009182	n/a
AK015236	n/a
AK016255	n/a
AK016672	n/a
AK035187	n/a
AK040197	n/a
AK133627	n/a
AK145544	n/a
AK166824	n/a
Amotl2	Mus musculus angiomotin-like 2 (Amotl2), mRNA.
Ankle1	Mus musculus ankyrin repeat and LEM domain containing 1 (Ankle1), transcript variant 2, mRNA.
Bag3	Mus musculus BCL2-associated athanogene 3 (Bag3), mRNA.
BC010587	n/a
BC116202	n/a
C130026I21Rik	n/a
Cnn3	Mus musculus calponin 3, acidic (Cnn3), mRNA.
Coq9	Mus musculus coenzyme Q9 (Coq9), mRNA.
D8Ertd738e	Mus musculus DNA segment, Chr 8, ERATO Doi 738, expressed (D8Ertd738e), mRNA.
Ddx5	Mus musculus DEAD (Asp-Glu-Ala-Asp) box polypeptide 5 (Ddx5), mRNA.
Dnajb1	Mus musculus DnaJ heat shock protein family (Hsp40) member B1 (Dnajb1), transcript variant 1, mRNA.
Dpp10	Mus musculus dipeptidylpeptidase 10 (Dpp10), mRNA.
Eef1a1	Mus musculus eukaryotic translation elongation factor 1 alpha 1 (Eef1a1), mRNA.
EF651816	n/a

Eif1	Mus musculus eukaryotic translation initiation factor 1 (Eif1), mRNA.
Eif4a2	Mus musculus eukaryotic translation initiation factor 4A2 (Eif4a2), transcript variant 4, non-coding RNA.
Erdr1	Mus musculus erythroid differentiation regulator 1 (Erdr1), transcript variant 1, coding, mRNA.
Esd	Mus musculus esterase D/formylglutathione hydrolase (Esd), transcript variant 1, mRNA.
Etfrf1	Mus musculus electron transfer flavoprotein regulatory factor 1 (Etfrf1), transcript variant 1, mRNA.
Fut10	Mus musculus fucosyltransferase 10 (Fut10), transcript variant 4, mRNA.
Fzd2	Mus musculus frizzled class receptor 2 (Fzd2), mRNA.
Gm10324	Mus musculus predicted gene 10324 (Gm10324), mRNA.
Gm15127	Mus musculus predicted gene 15127 (Gm15127), mRNA.
Gm15319	Mus musculus predicted gene 15319 (Gm15319), mRNA.
Gm21119	Mus musculus predicted gene, 21119 (Gm21119), mRNA.
Gm21190	Mus musculus predicted gene, 21190 (Gm21190), mRNA.
Gm3002	Mus musculus predicted gene 3002 (Gm3002), non-coding RNA.
Gm3020	Mus musculus predicted gene 3020 (Gm3020), non-coding RNA.
Gtpbp3	Mus musculus GTP binding protein 3 (Gtpbp3), mRNA.
H3f3b	Mus musculus H3 histone, family 3B (H3f3b), mRNA.
Hist1h1d	Mus musculus histone cluster 1, H1d (Hist1h1d), mRNA.
Hist1h1e	Mus musculus histone cluster 1, H1e (Hist1h1e), mRNA.
Hist2h2aa2	Mus musculus histone cluster 2, H2aa2 (Hist2h2aa2), mRNA.
Hist2h2ab	Mus musculus histone cluster 2, H2ab (Hist2h2ab), mRNA.
Hist2h2ac	Mus musculus histone cluster 2, H2ac (Hist2h2ac), mRNA.
Hist4h4	Mus musculus histone cluster 4, H4 (Hist4h4), mRNA.
Hmgb2	Mus musculus high mobility group box 2 (Hmgb2), mRNA.
Hnrnpa2b1	Mus musculus heterogeneous nuclear ribonucleoprotein A2/B1 (Hnrnpa2b1), transcript variant 1, mRNA.
Hnrnpa3	Mus musculus heterogeneous nuclear ribonucleoprotein A3 (Hnrnpa3), transcript variant c, mRNA.
Hnrnpm	Mus musculus heterogeneous nuclear ribonucleoprotein M (Hnrnpm), transcript variant 2, mRNA.
Insig1	Mus musculus insulin induced gene 1 (Insig1), mRNA.
Kras	Mus musculus Kirsten rat sarcoma viral oncogene homolog (Kras), mRNA.
Lias	Mus musculus lipoic acid synthetase (Lias), transcript variant 1, mRNA.
Malat1	Mus musculus metastasis associated lung adenocarcinoma transcript 1 (non-coding RNA) (Malat1), long non-coding RNA.

Mat2a	Mus musculus methionine adenosyltransferase II, alpha (Mat2a), mRNA.
Mgat4c	Mus musculus MGAT4 family, member C (Mgat4c), transcript variant 4, mRNA.
Mid1	Mus musculus midline 1 (Mid1), transcript variant 6, mRNA.
Mir6546	Mus musculus microRNA 6546 (Mir6546), microRNA.
Mmab	Mus musculus methylmalonic aciduria (cobalamin deficiency) cblB type homolog (human) (Mmab), transcript variant 1, mRNA.
Mphosph6	Mus musculus M phase phosphoprotein 6 (Mphosph6), transcript variant 1, mRNA.
Mrm3	Mus musculus mitochondrial rRNA methyltransferase 3 (Mrm3), mRNA.
Mrpl12	Mus musculus mitochondrial ribosomal protein L12 (Mrpl12), mRNA.
Mtus1	Mus musculus mitochondrial tumor suppressor 1 (Mtus1), transcript variant 3, mRNA.
Mup2	Mus musculus major urinary protein 2 (Mup2), transcript variant 2, mRNA.
Mup8	Mus musculus major urinary protein 8 (Mup8), mRNA.
Mup9	Mus musculus major urinary protein 9 (Mup9), mRNA.
Myl6	Mus musculus myosin, light polypeptide 6, alkali, smooth muscle and non-muscle (Myl6), transcript variant 2, mRNA.
Nbr1	Mus musculus neighbor of Brca1 gene 1 (Nbr1), transcript variant 2, mRNA.
Ndufb2	Mus musculus NADH dehydrogenase (ubiquinone) 1 beta subcomplex, 2 (Ndufb2), transcript variant 1, mRNA.
Nfe2l3	Mus musculus nuclear factor, erythroid derived 2, like 3 (Nfe2l3), mRNA.
Nkain2	Mus musculus Na+/K+ transporting ATPase interacting 2 (Nkain2), transcript variant 1, mRNA.
Npm3	Mus musculus nucleoplasmin 3 (Npm3), mRNA.
Opa1	Mus musculus OPA1, mitochondrial dynamin like GTPase (Opa1), transcript variant 2, mRNA.
Orc6	Mus musculus origin recognition complex, subunit 6 (Orc6), transcript variant 1, mRNA.
Particl	Mus musculus promoter of Mat2a antisense radiation induced circulating long non-coding RNA (Particl), long non-coding RNA.
Pcid2	Mus musculus PCI domain containing 2 (Pcid2), mRNA.
Pola2	Mus musculus polymerase (DNA directed), alpha 2 (Pola2), transcript variant 1, mRNA.
Polg2	Mus musculus polymerase (DNA directed), gamma 2, accessory subunit (Polg2), transcript variant 2, non-coding RNA.
Ranbp1	Mus musculus RAN binding protein 1 (Ranbp1), mRNA.
Rn4.5s	Mus musculus 4.5S RNA (Rn4.5s), ribosomal RNA.
Rn7sk	Mus musculus RNA, 7SK, nuclear (Rn7sk), small nuclear RNA.
Rnu2-10	Mus musculus U2 small nuclear RNA 10 (Rnu2-10), small nuclear RNA.

Rnu3b2	Mus musculus U3B small nuclear RNA 2 (Rnu3b2), small nuclear RNA.
Rpl27	Mus musculus ribosomal protein L27 (Rpl27), mRNA.
Rpl3	Mus musculus ribosomal protein L3 (Rpl3), mRNA.
Rpl36	Mus musculus ribosomal protein L36 (Rpl36), mRNA.
Rpl9	Mus musculus ribosomal protein L9 (Rpl9), mRNA.
Rpph1	Mus musculus ribonuclease P RNA component H1 (Rpph1), RNase P RNA.
Sart3	Mus musculus squamous cell carcinoma antigen recognized by T cells 3 (Sart3), transcript variant 2, mRNA.
Scfd1	Mus musculus Sec1 family domain containing 1 (Scfd1), transcript variant 1, mRNA.
Shcbp1	Mus musculus Shc SH2-domain binding protein 1 (Shcbp1), mRNA.
Slc35b1	Mus musculus solute carrier family 35, member B1 (Slc35b1), transcript variant 1, mRNA.
Snord2	Mus musculus small nucleolar RNA, C/D box 2 (Snord2), small nucleolar RNA.
Snord43	Mus musculus small nucleolar RNA, C/D box 43 (Snord43), small nucleolar RNA.
Speer4cos	Mus musculus spermatogenesis associated glutamate (E)-rich protein 4C, opposite strand transcript (Speer4cos), non-coding RNA.
Tcerg1	Mus musculus transcription elongation regulator 1 (CA150) (Tcerg1), transcript variant 3, non-coding RNA.
Tex14	Mus musculus testis expressed gene 14 (Tex14), transcript variant 2, mRNA.
Tinf2	Mus musculus Terf1 (TRF1)-interacting nuclear factor 2 (Tinf2), mRNA.
TRNA_Ala	transfer RNA Ala (anticodon AGC)
TRNA_Arg	transfer RNA Arg (anticodon CCT)
TRNA_Asn	transfer RNA Asn (anticodon GTT)
TRNA_Gly	transfer RNA Gly (anticodon GCC)
TRNA_Leu	transfer RNA Leu (anticodon AAG)
TRNA_Pro	transfer RNA Pro (anticodon AGG)
TRNA_Thr	transfer RNA Thr (anticodon TGT)
TRNA_Tyr	transfer RNA Tyr (anticodon GTA)
Tsen15	Mus musculus tRNA splicing endonuclease subunit 15 (Tsen15), mRNA.
Uba52	Mus musculus ubiquitin A-52 residue ribosomal protein fusion product 1 (Uba52), transcript variant 1, mRNA.
Ubc	Mus musculus ubiquitin C (Ubc), mRNA.
Ube2cbp	Mus musculus ubiquitin-conjugating enzyme E2C binding protein (Ube2cbp), mRNA.
Utp3	Mus musculus UTP3 small subunit processome component (Utp3), mRNA.

Vps33a	Mus musculus VPS33A CORVET/HOPS core subunit (Vps33a), transcript variant 1, mRNA.
Wdr45b	Mus musculus WD repeat domain 45B (Wdr45b), mRNA.
Yeats2	Mus musculus YEATS domain containing 2 (Yeats2), transcript variant 3, mRNA.
